# c-MET-positive circulating tumor cells and cell-free DNA as independent prognostic factors in hormone receptor-positive/HER2-negative metastatic breast cancer

**DOI:** 10.1186/s13058-024-01768-y

**Published:** 2024-01-18

**Authors:** Jieun Park, Eun Sol Chang, Ji-Yeon Kim, Chaithanya Chelakkot, Minjung Sung, Ji-Young Song, Kyungsoo Jung, Ji Hye Lee, Jun Young Choi, Na Young Kim, Hyegyeong Lee, Mi-Ran Kang, Mi Jeong Kwon, Young Kee Shin, Yeon Hee Park, Yoon-La Choi

**Affiliations:** 1 Department of Molecular Medicine and Biopharmaceutical Sciences, Graduate School of Convergence Science and Technology, Seoul National University, Seoul, 08826 Republic of Korea; 2 Department of Health Sciences and Technology, SAIHST, Sungkyunkwan University, Seoul, Republic of Korea; 3 Laboratory of Molecular Pathology and Theranostics, Samsung Medical Center, Sungkyunkwan University School of Medicine, Seoul, Republic of Korea; 4 Division of Hematology-Oncology, Department of Medicine, Samsung Medical Center, Sungkyunkwan University School of Medicine, Irwon-ro 81, Gangnam-gu, Seoul, 06351 Republic of Korea; 5 Technical Research Center, Genobio Corp., Seoul, Republic of Korea; 6 Laboratory of Molecular Pathology and Cancer Genomics, College of Pharmacy and Research Institute of Pharmaceutical Sciences, Seoul National University, Seoul, Republic of Korea; 7 Department of Pathology and Translational Genomics, Samsung Medical Center, Sungkyunkwan University School of Medicine, Irwon-ro 81, Gangnam-gu, Seoul, 06351 Republic of Korea; 8 R&D Center, ABION Inc., Seoul, Republic of Korea; 9 Central Laboratory, LOGONE Bio-Convergence Research Foundation, Seoul, Republic of Korea; 10 R&D Center, Gencurix Inc., Seoul, Republic of Korea; 11 Vessel-Organ Interaction Research Center (MRC), College of Pharmacy, Kyungpook National University, Daegu, Republic of Korea; 12 BK21 FOUR Community-Based Intelligent Novel Drug Discovery Education Unit, College of Pharmacy and Research Institute of Pharmaceutical Sciences, Kyungpook National University, Daegu, Republic of Korea

**Keywords:** Metastatic breast cancer, Circulating tumor cells, Prognostic biomarkers, c-MET, Cell-free DNA

## Abstract

**Background:**

Endocrine therapy resistance in hormone receptor-positive/HER2-negative (HR+/HER2−) breast cancer (BC) is a significant clinical challenge that poses several unmet needs in the management of the disease. This study aimed to investigate the prognostic value of c-MET-positive circulating tumor cells (cMET+ CTCs), *ESR1*/*PIK3CA* mutations, and cell-free DNA (cfDNA) concentrations in patients with hormone receptor-positive (HR+) metastatic breast cancer (mBC).

**Methods:**

Ninety-seven patients with HR+ mBC were prospectively enrolled during standard treatment at Samsung Medical Center. CTCs were isolated from blood using GenoCTC^®^ and EpCAM or c-MET CTC isolation kits. *PIK3CA* and *ESR1* hotspot mutations were analyzed using droplet digital PCR. CfDNA concentrations were calculated using internal control copies from the *ESR1* mutation test. Immunocytochemistry was performed to compare c-MET overexpression between primary and metastatic sites.

**Results:**

The proportion of c-MET overexpression was significantly higher in metastatic sites than in primary sites (*p* = 0.00002). Survival analysis showed that c-MET+ CTC, cfDNA concentration, and *ESR1* mutations were significantly associated with poor prognosis (*p* = 0.0026, 0.0021, and 0.0064, respectively) in HR+/HER2− mBC. By contrast, EpCAM-positive CTC (EpCAM+ CTC) and *PIK3CA* mutations were not associated with progression-free survival (PFS) in HR+/HER2− mBC. Multivariate analyses revealed that c-MET+ CTCs and cfDNA concentration were independent predictors of PFS in HR+/HER2− mBC.

**Conclusions:**

Monitoring c-MET+ CTC, rather than assessing c-MET expression in the primary BC site, could provide valuable information for predicting disease progression, as c-MET expression can change during treatment. The c-MET+ CTC count and cfDNA concentration could provide complementary information on disease progression in HR+ /HER2− mBC, highlighting the importance of integrated liquid biopsy.

**Supplementary Information:**

The online version contains supplementary material available at 10.1186/s13058-024-01768-y.

## Background

Hormone receptor-positive (HR+) breast cancer (BC) accounts for more than 65–70% of BC cases, and four fifths of these are HR+/HER2-negative (HER2−). Endocrine resistance and late recurrence are major clinical concerns in patients with HR+ BC. Although the availability of effective endocine therapy has significantly improved survival rates, approximately 25–30% of patients develop primary or secondary endocrine resistance owing to intrinsic or acquired mechanisms [1]. Updated guidelines for the treatment of advanced BC suggest that cyclin-dependent kinase 4 and 6 inhibitors (CDK4/6i) in combination with endocrine therapy improve the overall survival (OS) of patients; however, most patients eventually develop acquired drug resistance to CDK4/6i [2]. Several factors contribute to endocrine resistance, including genetic and epigenetic alterations in the estrogen receptor (ER)/progesterone receptor (PR) pathway, activation of the phosphatidylinositol 3-kinase (PI3K)/mTOR pathway, and HER2 reactivation or acquired mutations of *HER2* [3]. Therefore, the development of novel biomarker assays to predict the occurrence of endocrine resistance or disease progression is essential for improving patient outcomes.

Liquid biopsy (LBx) provide real-time molecular profiling of cancer, and numerous studies have shown that LBx analytes are valuable biomarkers for the diagnosis, prognosis, prediction, and recurrence monitoring of various cancers [4]. Circulating tumor cell (CTC) and cell-free (cf) circulating tumor DNA (ctDNA) are widely studied cancer-derived components in patients’ blood. CTCs play an essential role in metastasis and are strongly correlated with progression-free survival (PFS) and OS of cancer patients [5–7]. US Food and Drug Administration (FDA) has approved CellSearch^®^ system and Parsortix™ PC1 system. Surface marker-dependent CellSearch^®^ system enumerates epithelial cellular adhesion molecule (EpCAM) + CTCs, but not all CTCs express EpCAM [8]. Moreover, the mesenchymal phenotype of CTCs has been implicated in poor prognosis [9, 10]. Parsortix™ PC1 system, agnostic of cell surface biomarker, enriches CTCs with a certain size and deformability including mesenchymal CTCs and CTC clusters [11].

Targeted next-generation sequencing and droplet digital polymerase chain reaction (ddPCR)-based mutational analysis precisely detect mutations indicative of endocrine therapy resistance, aiding in treatment decision [12, 13]. Acquired mutations in *ESR1* have been identified as a frequent driver of endocrine therapy resistance in HR+ mBC, especially in patients treated with aromatase inhibitor (AI), accounting for approximately 20% of recurrent cases [14]. Randomized clinical trials, including SOFeA, PALOMA3, and FERGI, have identified *ESR1* mutations in a significant proportion (approximately 28–39%) of patients with HR+/HER2− mBC [15, 16]. Aberrations in the expression of targetable molecular biomarkers between primary and metastatic/recurrent tumors have been widely reported [17], and the findings from these studies highlight the importance of monitoring these biomarkers to guide therapy decision [18]. Recently, routine testing of *ESR1* mutations at recurrence or progression on endocrine therapy in patients with ER+/HER2− mBC were strongly recommended [19].

MET (MNNG-HOS transforming gene) encodes receptor tyrosine kinase c-MET, which is essential for cell proliferation, morphogenesis, and wound healing. Hepatocyte growth factor (HGF) induces c-MET dimerization and autophosphorylation, activating various signal transduction pathways including the mitogen-activated protein kinase (MAPK)/PI3K pathway for survival, migration, angiogenesis, and stemness [20]. The HGF/c-MET signaling pathway may be involved in various cellular processes, including carcinogenesis, proliferation, survival, metastasis, epithelial-mesenchymal transition (EMT), and drug resistance in cancer cells [21].

An association between c-MET alterations and drug resistance has been reported previously. In patients with HR+ mBC treated with exemestane plus everolimus, c-MET overexpression was reportedly associated with shorter PFS and higher frequency of visceral metastases [22]. In a phase II study nextMONARCH 1, 8% of patients with HR+/HER2− advanced BC treated with abemaciclib plus tamoxifen showed new *MET* genetic alterations that were potentially associated with drug resistance [23]. Patients with advanced gastric cancer reportedly show alterations in c-MET expression after chemotherapy and worse outcomes in the c-MET+ group [24]. Preclinical studies have demonstrated the association between c-MET signaling and chemoresistance [25].

Therefore, we hypothesized that c-MET-positive (c-MET+) CTCs may be associated with poor prognosis in patients with HR+ mBC. To address the prognostic impact of c-MET+ CTCs, we developed a c-MET+ CTC-detection assay. These assays were applied in a prospective study to investigate the prognostic value of LBx analytes in patients with HR+ mBC. This study aimed to evaluate whether c-MET+ CTCs can be detected in the blood and whether c-MET+ CTCs and cfDNA are predictors of disease progression in patients with HR+ mBC.

## Materials and methods

### Study design

This prospective, partially blinded, single-center study included 97 patients with HR+ mBC to investigate the prognostic impact of LBx analytes including cMET+ CTC in patients with HR+ mBC. Patients were recruited during their standard treatment course at Samsung Medical Center (SMC), Seoul, Republic of Korea, between May and December 2020. The study protocol was approved by the Institutional Review Board (IRB) of the SMC (IRB No. 2019-08-119) and was conducted per the Declaration of Helsinki. Written informed consent was obtained from all the patients. During treatment, peripheral blood samples were collected once using cell-free DNA BCT (Streck, La Vista, NE, USA), and a volume of 20 mL was obtained. Patient characteristics and tumor histology details were extracted from the pathology reports at the Department of Pathology, SMC. Disease progression was evaluated using radiography images based on the Response Evaluation Criteria in Solid Tumors (RECIST, version 1.1) guideline [26]. PFS was defined as the time from blood collection to radiological disease progression or death from any cause.

### *Evaluating the analytical performance of the c-MET* + *CTC assay using spiking*

The human BC cell line (MCF7) and human gastric cancer cell line (SNU5) were obtained from the Korean Cell Line Bank (KCLB, Seoul, Republic of Korea). Cell lines were authenticated by short tandem repeat profiling analyses in Korea Genome Information Institute, and the e-Myco VALID Mycoplasma PCR detection kit (iNtRon Biotechnology, Inc., Seongnam-si, Republic of Korea; Cat#25245) was used to verify that the cells were not contaminated with mycoplasma. The cells were maintained in RPMI-1640 culture medium supplemented with 10% fetal bovine serum, 100 U/mL penicillin, and 100 µg/mL streptomycin (Gibco, Rockville, MD, USA; Cat#15140122) at 37 °C in a 5% humidified CO_2_ incubator. To evaluate the c-MET+ CTC isolation method, SNU5 and MCF7 cells were spiked into RPMI-1640 culture medium or healthy human blood samples, which were obtained with written informed consent under IRB approval from the SMC (IRB No. 2021-08-063). Briefly, cells were spiked into 1 mL of the cell culture medium or healthy human blood samples. Cells were incubated with anti-c-MET monoclonal antibodies conjugated to magnetic beads and isolated as described in the c-MET CTC isolation kit using GenoCTC^®^ (Genobio Corp, Seoul, Republic of Korea). Detailed methods for the spiking experiments are provided in the Additional file 1. The recovery and separation rates were calculated using the following equations:

$${\text{Recovery}}\;{\text{rate}}\left( \% \right) = \frac{{{\text{Output}}\;{\text{cells}}\left( {{\text{Waste}} + {\text{collected}}\;{\text{cells}}} \right)}}{{{\text{Total}}\;{\text{input}}\;{\text{cells}}}} \times 100$$

$${\text{Separation}}\;{\text{rate}}\left( \% \right) = \frac{{{\text{Collected}}\;{\text{cells}}}}{{{\text{Output}}\;{\text{cells}}\left( {{\text{Waste}} + {\text{collected}}\;{\text{cells}}} \right)}} \times 100$$

### EpCAM + or c-MET + CTC isolation and multi-color immunocytochemical analysis

All blood samples were processed within four days of the blood draw, with 73% of the samples processed within one day. CTCs were isolated using GenoCTC^®^ (Genobio Corp), an immune magnetophoretic CTC isolation device, according to a previously published protocol [27]. Briefly, 4 mL of blood was incubated with anti-c-MET or anti-EpCAM monoclonal antibodies conjugated to magnetic beads, which are components of EpCAM or c-MET CTC isolation kits (Genobio Corp), for 30 min at room temperature. After incubation with the reagents, the samples were loaded onto the GenoCTC^®^ device and CTC isolation was performed.

CTCs collected from the GenoCTC^®^ device were centrifuged at 1000 rpm for 5 min. The supernatant was removed and approximately 10 µL of the sample was maintained for slide preparation. The CTC suspension and isolated peripheral blood mononuclear cells (PBMCs) were gently placed on glass slides and dried in a hybridizer (Dako Colarado Inc., Fort Collins, CO, USA) at 37 °C. PBMCs were isolated from 1 mL of blood using LymphoPrep™ (STEMCELL Technologies Inc., Vancouver, British Columbia, Canada) as described by the manufacturer. The cells were stained using a GenoCTC profiling kit (Genobio Corp), as described previously [27]. The cells were visualized using a Nikon Eclipse microscope equipped with an Infinity# camera (Nikon Eclinpse Inc., Tokyo, Japan), and the images were analyzed and enumerated using GenoAnalyzer v1.0 software (Genobio Corp). CTCs were defined as DAPI+, CK-18+, or CD45-. PBMC slides were used as the CD45+ slides. CTC status of patients was determined as high and low based on the CTC counts in 4 mL blood exhibiting maximal statistical significance in PFS of HR+ mBC. EpCAM+ CTC was considered high if ≥ 4 CTCs/4 mL blood and low if < 4 CTCs/4 mL blood, while c-MET+ CTC was high if ≥ 3 CTCs/4 mL blood and low if < 3 CTCs/4 mL blood.

### Isolation and analysis of cfDNA from PB samples

Blood samples were centrifuged at 3000× *g* for 15 min at 4 °C to separate plasma. cfDNA was extracted from 4 to 6 mL plasma samples using the QIAamp Circulating Nucleic Acid Kit (Qiagen, Hilden, Germany) according to the manufacturer’s protocol. Circulating nucleic acids were eluted in 100 µL of elution buffer. *ESR1* and *PIK3CA* mutations in cfDNA samples were analyzed using Droplex PIK3CA and Droplex ESR1 Mutation Test Kits (Gencurix Inc., Seoul, Republic of Korea) according to the manufacturer’s protocol. Briefly, ddPCR reagents were mixed with 12.9 µL eluted cfDNA/well in an 8-strip PCR tube. Primers and probe sets were designed to detect mutations in *PIK3CA* hotspot mutations (R88Q, N345K, E542, E545, Q546, E726, H1047, M1048, G1049) and *ESR1* hotspot mutations (E380, S463, V534, L536, Y537, and D538). Positive and negative controls were mixed with the reaction mixture and placed in an 8-strip PCR tube. This mixture was entered into a QX200TM Droplet Generator (Bio-Rad, Hercules, CA, USA) and turned into droplets. The droplets were subjected to PCR in 96-well plates. After amplification, the droplets were counted using a QX200TM Droplet Reader (Bio-Rad). An internal control (IC) was designed to detect *PIK3CA* or *ESR1* and was used as an indicator of the cfDNA concentration and mutation index. cfDNA concentration and mutation index were calculated as follows:

$$\begin{aligned} {\text{cfDNA}}\;{\text{concentrations}}/{\text{mL}}\;{\text{plasma}} & = {\text{IC}}\;{\text{copies}}/{\text{well}} \times \frac{{{\text{total}}\;{\text{DNA}}\;{\text{elution}}\;{\text{volume}}}}{{{\text{DNA}}\;{\text{loading}}\;{\text{volume/well}}}} \\ & \quad \times \frac{1}{{{\text{plasma}}\;{\text{volume}}\left( {{\text{mL}}} \right)}} \\ \end{aligned}$$

$${\text{Mutation}}\;{\text{Index}} = \frac{{{\text{Mutant}}\;{\text{copies}}\;{\text{of}}\;{\text{PIK}}3{\text{CA}}\;{\text{or}}\;{\text{ESR}}1}}{{{\text{Total}}\;{\text{copies}}\;{\text{of}}\;{\text{PIK}}3{\text{CA}}\;{\text{or}}\;{\text{ESR}}1}} \times 100\%$$

The cfDNA concentration was calculated using IC copies from the Droplex ESR1 Mutation Test Kits. Patients were classified as having high or low cfDNA concentration using a cut-off value of 1490 copies/mL plasma, which was determined to have maximal statistical significance in PFS of HR+ mBC.

### Analysis of c-MET expression in primary and metastatic sites of BC

Patients with primary BC (*n* = 980) were enrolled from the Breast Cancer Biomarker Study (BCBS) tissue microarray (TMA) cohort [28, 29], which comprised Korean patients with BC who did not receive cytotoxic chemotherapy or anti-HER2 therapy. The SMC IRB approved this study to determine the c-MET expression levels in this cohort (IRB 2020-09-119). The loss of tumor samples or clinicopathological data resulted in the exclusion of 260 patients. ER and PR statuses were defined based on pathology reports at the Department of Pathology, SMC, and HER2 status was independently scored as described in a previous study [28]. Twenty-seven metastatic sites in HR+/HER2− mBC were independently collected to evaluate c-MET overexpression. All clinicopathological data were anonymized and de-identified prior to the analysis. c-MET expression was evaluated by immunohistochemistry (IHC) using anti-Total c-MET (SP44) rabbit monoclonal antibodies (Ventana Medical Systems, Tucson, AZ, USA) and a Ventana Discovery XY automated system (Ventana Medical Systems) according to the manufacturer's instructions. Membranous staining was scored as follows: 0, no reactivity; 1+, weak or moderate staining in < 50% of tumor cells; 2+, weak staining in ≥ 50% of tumor cells or strong intensity in > 10% of tumor cells; and 3+, strong staining in ≥ 50% of tumor cells. c-MET was considered positive if staining was scored as 2+ or 3+.

### Statistical analysis

Associations between categorical variables were assessed using chi-square or Fisher’s exact test. All data were expressed as mean ± standard deviation. For continuous variables, we performed an unpaired *t*-test between two groups. A log-rank test was used to estimate the Kaplan–Meier curve and compare PFS. Cut-off values were calculated using the maxstat R package, which estimates cut-off values based on standardized log-rank statistics. Univariate and multivariate analyses were performed using the Cox proportional hazard regression model. All hazard ratios are reported with 95% confidence intervals (CIs). *P* < 0.05 was considered statistically significant. Statistical analyses were performed using R Studio version 1.4.1103 or GraphPad Prism version 9.3.1 software.

## Results

### Patient characteristics

Ninety-seven patients with HR+ mBC were enrolled in the study, with four exclusions owing to blood cell contamination during CTC isolation. The pathological and clinical characteristics of patients are shown in Table 1. The median age of the enrolled patients at the time of diagnosis was 45 years (range, 29–65 years). All patients had invasive ductal carcinoma, except for one patient with unavailable histology. Among the included patients, 69% (63/93) and 32% (30/93) were classified as having HR+/HER2− and HR+/HER2+ mBCs, respectively. High c-MET+ CTC counts were significantly associated with HER2 status in patients with HR+ mBCs (*p* = 0.02). Low cfDNA concentration was correlated with first line of palliative therapy (*p* < 0.001). The time intervals between treatment initiation and blood draw were not significant when grouped by c-MET+ CTC or EpCAM+ CTC status, or cfDNA concentration in HR+/HER2− or HR+/HER2+ mBC (Additional file 2: Fig. S1).

**Table 1:** Patient characteristics

	Total	c-MET+ CTC			EpCAM+ CTC			cfDNA concentration		
		Low (< 3)	High (≥ 3)	*P* value	Low (< 4)	High (≥ 4)	*P* value	Low (< 1490)	High (≥ 1490)	*P* value
*Age at diagnosis, n(%)*										
Median (range)	45 (29–65)	46 (29–65)	43 (32–59)		46 (29–65)	43 (33–63)		45 (29–63)	43 (31–65)	
50<	60	48 (61.5)	12 (80.0)	0.28	48 (64.0)	12 (66.7)	1.00	33 (66.0)	27 (62.8)	0.92
50≥	33	30 (38.5)	3 (20.0)		27 (36.0)	6 (33.3)		17 (34.0)	16 (37.2)	
*Primary tumor subtype, n(%)*										
HR+/HER2−	63	57 (73.1)	6 (40.0)	0.02	51 (68.0)	12 (66.7)	1.00	34 (68.0)	29 (67.4)	1.00
HR+/HER2+	30	21 (26.9)	9 (60.0)		24 (32.0)	6 (33.3)		16 (32.0)	14 (32.6)	
*Ki67 status (cut point* = *20%), n(%)*										
Low	29	25 (50.0)	4 (33.3)	0.47	26 (46.4)	3 (50.0)	1.00	15 (53.6)	14 (41.2)	0.47
High	33	25 (50.0)	8 (66.7)		30 (53.6)	3 (50.0)		13 (46.4)	20 (58.8)	
NA	31	28	3		19	12		22	9	
*Histologic grade, n(%)*										
1	7	6 (8.3)	1 (8.3)	0.27	4 (5.9)	3 (18.8)	0.21	4 (9.3)	3 (7.3)	0.35
2	56	50 (69.4)	6 (50.0)		47 (69.1)	9 (56.3)		31 (72.1)	25 (61.0)	
3	21	16 (22.2)	5 (41.7)		17 (25.0)	4 (25.0)		8 (18.6)	13 (31.7)	
NA	9	6	3		7	2		7	2	
*Nuclear grade, n(%)*										
1	4	4 (5.6)	0 (0.0)	0.35	3 (4.5)	1 (5.9)	1.00	2 (4.7)	2 (4.9)	0.09
2	58	51 (70.8)	7 (58.3)		46 (68.7)	12 (70.6)		34 (79.1)	24 (58.5)	
3	22	17 (23.6)	5 (41.7)		18 (26.9)	4 (23.5)		7 (16.3)	15 (36.6)	
NA	9	6	3		8	1		7	2	
*ECOG performance score, n(%)*										
0	19	17 (22.1)	2 (13.3)	0.73	15 (20.3)	4 (22.2)	1.00	8 (16.3)	11 (25.6)	0.40
1	73	60 (77.9)	13 (86.7)		59 (79.7)	14 (77.8)		41 (83.7)	32 (74.4)	
NA	1	22	0		22	0		1	0	
*Metastatic sites, n(%)*										
Visceral	59	49 (62.8)	10 (66.7)	0.99	48 (64.0)	11 (61.1)	0.86	30 (60.0)	29 (67.4)	0.68
Bone only	13	11 (14.1)	2 (13.3)		11 (14.7)	2 (11.1)		7 (14.0)	6 (14.0)	
Others	21	18 (23.1)	3 (20.0)		16 (21.3)	5 (27.8)		13 (26.0)	8 (18.6)	
*Line of palliative therapy, n(%)*										
1	37	30 (38.5)	7 (46.7)	0.88	27(36.0)	10 (55.6)	0.25	30 (60.0)	7 (16.3)	< 0.001
2	22	19 (24.4)	3 (20.0)		20 (26.7)	2 (11.1)		7 (14.0)	15 (34.9)	
≥ 3	34	29 (37.2)	5 (33.3)		28 (37.3)	6 (33.3)		13 (26.0)	21 (48.8)	
*Treatment, n(%)*										
Cytotoxic chemotherapy	35	32 (41.0)	3 (20.0)	0.32	28 (35.3)	7 (38.9)	0.61	17 (34.0)	18 (41.9)	0.33
Targeted agents	18	14 (17.9)	4 (26.7)		16 (21.3)	2 (11.1)		7 (14.0)	11 (25.6)	
Endocrine therapy only	5	5 (6.4)	0 (0.0)		5 (6.7)	0 (0.0)		3 (6.0)	2 (4.7)	
Endocrine therapy + CDK4/6i	31	24 (30.8)	7 (46.7)		23 (30.7)	8 (44.4)		21 (42.0)	10 (23.3)	
Endocrine therapy + Targeted agents	4	3 (3.8)	1 (6.7)		3 (4.0)	1 (5.6)		2 (4.0)	2 (4.7)	
*Previous endocrine therapy, n(%)*										
Yes	76	65 (83.3)	11 (73.3)	0.46	61 (82.4)	15 (83.3)	1.00	43 (87.8)	33 (76.7)	0.27
No	17	13 (16.7)	4 (26.7)		13 (17.6)	3 (16.7)		6 (12.2)	10 (23.3)	
*Previous chemotherapy in any setting, n(%)*										
Yes	77	63 (80.8)	14 (93.3)	0.45	61 (81.3)	16 (88.9)	0.73	39 (78.0)	38 (88.4)	0.30
No	16	15 (19.2)	1 (6.7)		14 (18.7)	2 (11.1)		11 (22.0)	5 (11.6)	

*HR* hormone receptor, *ECOG* Eastern Cooporative Oncology Group, *CTC* circulating tumor cell, *cfDNA* cell-free DNA

### Isolation and enumeration results of EpCAM+ or c-MET+ CTCs

Analytical performance data of the c-MET+ CTC assay used in the study are presented in Additional file 3: Fig. S2. For SNU5 cells spiked into culture media or healthy blood, the recovery rates were 58.7 ± 0.5% or 70.6 ± 5.5% (spiked in 10,000 cells) and 61.0 ± 9.2% or 64.7 ± 11.8% (spiked in 1000–3000 cells), respectively, and the separation rates were 73.4 ± 6.7% or 81.3 ± 13.6% (spiked in 10,000 cells) and 67.7 ± 7.1% or 85.3 ± 11.6% (spiked in 1000–3000 cells), respectively. For MCF7 cells which had a minimal c-MET expression, the recovery rates were 80.3 ± 7.8% (spiked in 10,000 cells) and 76.7 ± 11.2% (spiked in 1000–3000 cells); however, the separation rates were 2.0 ± 3.5% (spiked in 10,000 cells) and 0.0 ± 0.0% (spiked in 1000–3000 cells), respectively.

**Fig. 1:**
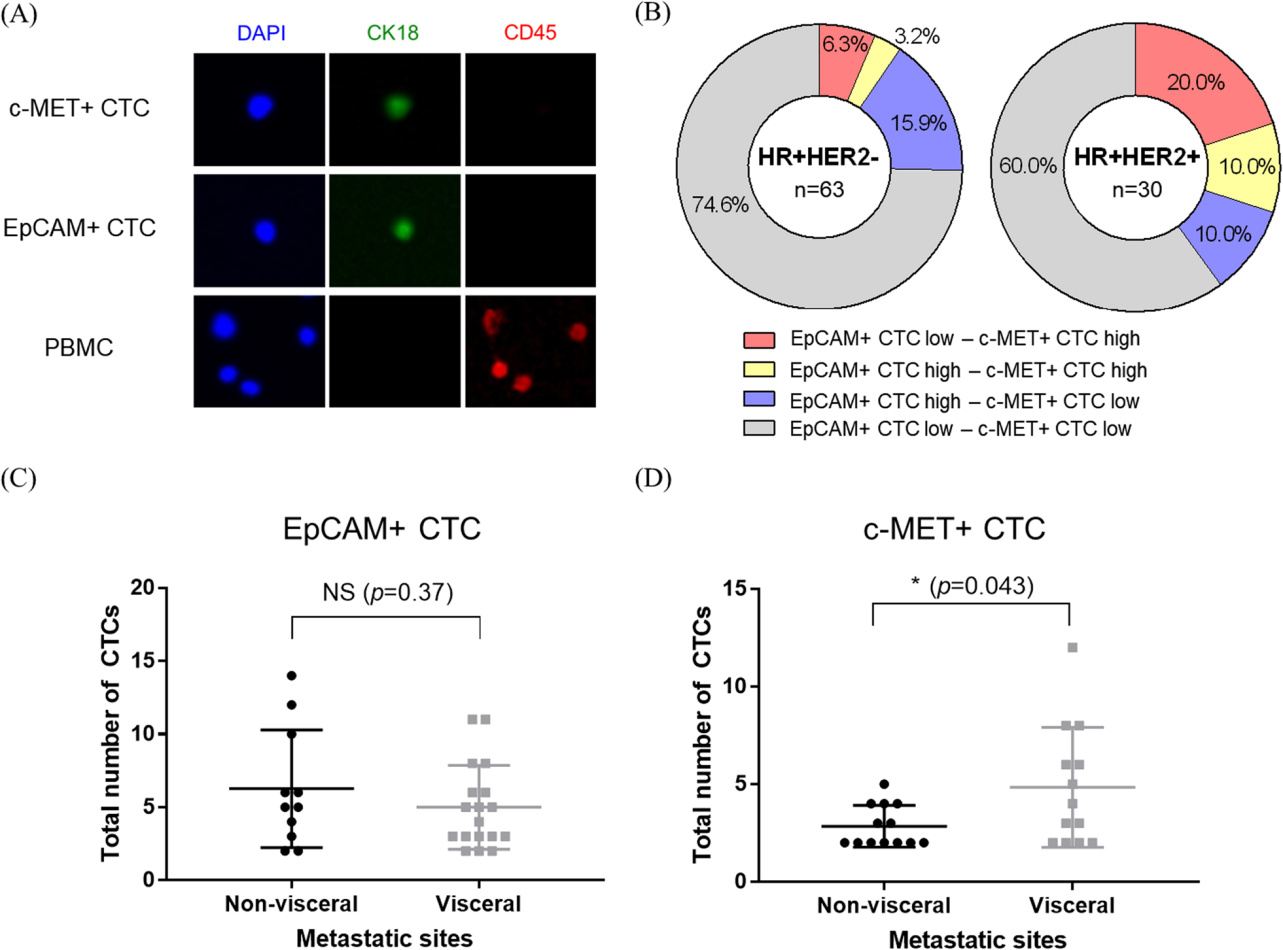
CTC enumeration results of patients with HR+ mBC. **A** Representative images of c-MET- or EpCAM+ CTC. CTCs were independently captured using anti-EpCAM or anti-c-MET antibody and defined as DAPI+, CK18+, and CD45−. **B** Proportions of high EpCAM+ or c-MET+ CTCs group by HER2 status. EpCAM+ CTC high represents four or more CTCs detected in 4 mL blood, and c-MET+ CTC high represents three or more CTCs detected in 4 mL blood. The number of EpCAM+ **C** or c-MET+ **D** CTCs in patients with detected CTCs by the presence of visceral metastasis. PBMC, peripheral blood mononuclear cell; HR, hormone receptor; mBC, metastatic breast cancer; CTCs, circulating tumor cells; *p < 0.05

Representative images of EpCAM+ and c-MET+ CTCs are shown in Fig. 1A. Among the included patients, 46.2% (43/93) had more than one EpCAM+ or c-MET+ CTCs, 31.2% (29/93) had EpCAM+ CTCs, 27.9% (26/93) had c-MET+ CTCs, and 12.9% (12/93) had both EpCAM+ and c-MET+ CTCs in their PB samples. In patients with HR+ /HER2− mBC, 19.1% had EpCAM+ CTC high (≥ 4 CTCs/4 mL blood) and 9.5% had c-MET+ CTC high (≥ 3 CTCs/4 mL blood). In patients with HR+ /HER2+ mBC, 20% had EpCAM+ CTC high and 30% had c-MET+ CTC high (Fig. 1B). There was a significant difference (*p* = 0.043) in the number of c-MET+ CTCs between patients with visceral metastasis and those with non-visceral metastasis. Subgroup analysis with visceral metastasis had shown that patients with liver metastasis exhibited a significant difference (*p* = 0.019) in the number of c-MET+ CTCs when compared patients with non-visceral metastasis (Additional file 4: Fig S3). However, no correlation was observed between these groups in EpCAM+ CTCs (*p* = 0.37) (Fig. 1C, D).

### Prognostic values of EpCAM+ or c-MET+ CTCs in patients with HR+ mBC

**Fig. 2:**
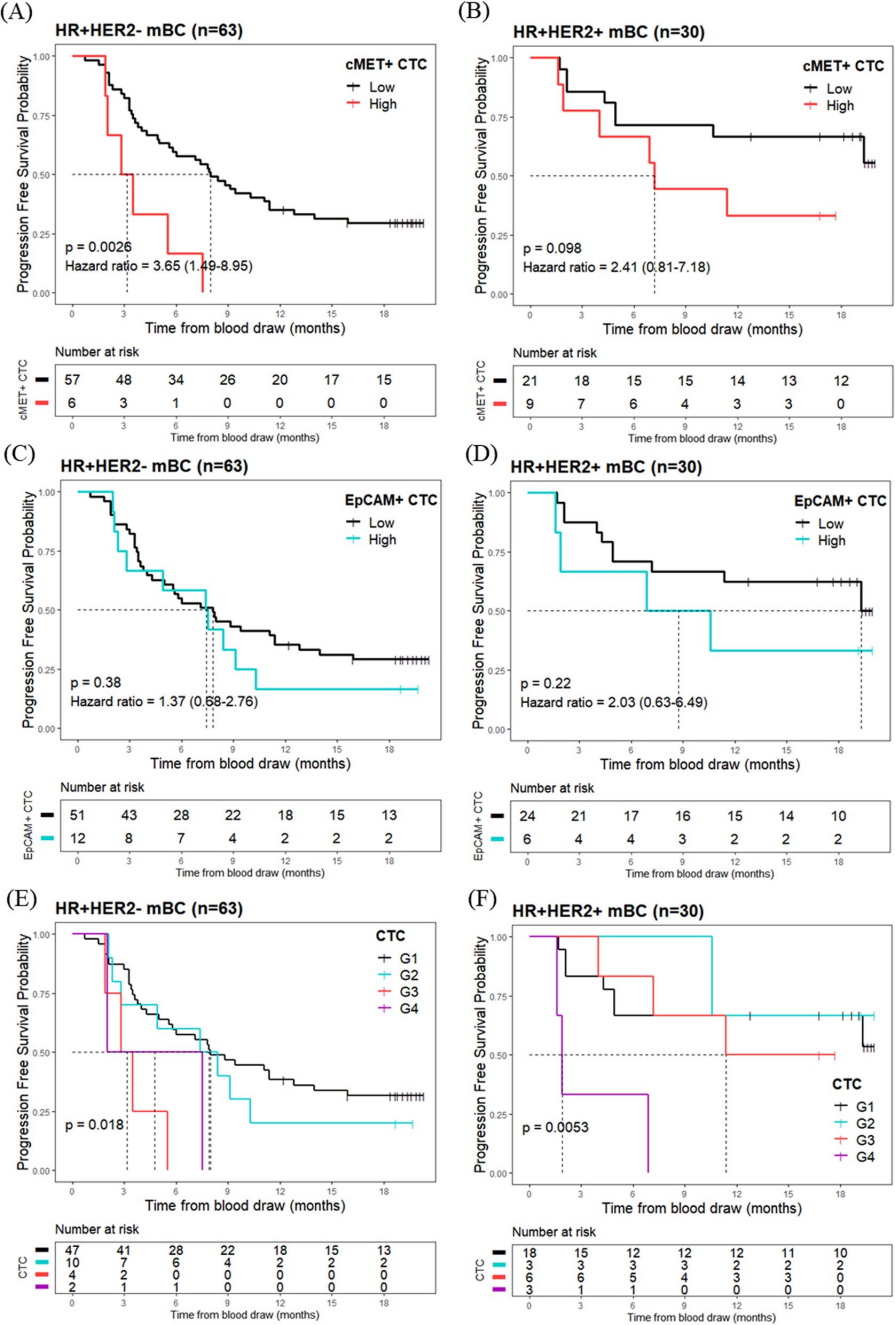
Progression-free survival (PFS) analysis based on EpCAM+ or c-MET+ CTC count. Kaplan–Meier curves of PFS according to the level of c-MET+ CTC in **A** HR+/HER2−, **B** HR+/HER2+, or EpCAM+ CTC in patients with **C** HR+/HER2− and **D** HR+/HER2+ mBC. For combined analysis of the EpCAM+ and c-MET+ CTC, patients were classified into four groups: c-MET+ CTC low/EpCAM+ CTC low (G1), c-MET+ CTC low/EpCAM+ CTC high (G2), c-MET+ CTC high/EpCAM+ CTC low (G3), and c-MET+ CTC high/EpCAM+ CTC high (G4) in HR+ /HER2− (**E**) or HR+/HER− (**F**). PFS was calculated as the time from blood draw to either disease progression or death during standard therapy. *CTCs, circulating tumor cells; HR, hormone receptor; mBC, metastatic breast cancer*

At the data cut-off (February 10, 2022), 64.5% of patients showed disease progression. The median follow-up time was 8.4 months (min–max, 0.7–20.3), and the median time to censoring was 18.7 months (min–max, 12.2–20.3). The c-MET+ CTC high group had shorter PFS (median PFS = 3.2 months, 95% CI 2.0–not estimable) than those in the c-MET+ CTC low group (median PFS = 8.0 months, 95% CI 5.6–11.4) (hazard ratio = 3.7, 95% CI 1.5–9.0, *p* = 0.0026) in HR+/HER2− mBC (Fig. 2A). However, no statistical significance was achieved between the c-MET+ CTC high (median PFS = 7.2, 95% CI 4.0–not estimable) and the c-MET+ CTC low group (median PFS = NA, 95% CI 10.6–not estimable) (hazard ratio = 2.4, 95% CI 0.8–7.2, *p* = 0.098) in HR+/HER2+ mBC (Fig. 2B). EpCAM+ CTC high groups did not correlate with shorter PFS in both the HR+/HER2− (hazard ratio = 1.4, 95% CI 0.7–2.8) (Fig. 2C) and HR+/HER2+ mBC (hazard ratio = 2.0, 95% CI 0.6–6.5) (Fig. 2D).

For the combined analysis of EpCAM+ and c-MET+ CTCs, patients with HR+/HER2− and HR+/HER2+ were classified into four groups: c-MET+ CTC low/EpCAM+ CTC low (G1), c-MET+ CTC low/EpCAM+ CTC high (G2), c-MET+ CTC high/EpCAM+ CTC low (G3), and c-MET+ CTC high/EpCAM+ CTC high (G4). In patients with HR+/HER2−, G3 group was associated with poor prognosis (hazard ratio = 4.5, 95% CI 1.5–13.3, *p* = 0.0071), whereas G4 group was not significantly associated with PFS (hazard ratio = 3.1, 95% CI 0.7–13.1, *p* = 0.13) (Fig. 2E). Conversely, in patients with HR+/HER2+ status, the G4 group was significantly associated with reduced PFS (hazard ratio = 8.2, 95% CI 1.9–35.0, *p* = 0.0045), whereas the G3 group was not associated with poor PFS (hazard ratio = 1.4, 95% CI 0.3–5.4, *p* = 0.67) (Fig. 2F).

### Frequency of the c-MET overexpression in primary and metastatic sites of BC

Clinicopathological factors were not related to c-MET overexpression in either primary BC (*n* = 358) or mBC (*n* = 27) of HR+/HER2−, as indicated in Additional file 5: Table S4. Additional file 6: Fig. S5A shows the representative IHC staining intensities for c-MET expression. c-MET overexpression was observed in 4.7% (17/358), 3.8% (4/104), 7.1% (7/98), and 13.6% (22/162) of the primary sites in HR+/HER2−, HR+/HER2+, HER2-enriched, and Triple-negative breast cancer samples, respectively (Additional file 7: Table S6). In HR+ HER2− mBC samples, 22.2% (6/27) were c-MET-overexpressing tumors (Additional file 6: Fig. S5B, Table S6). Significant differences were observed in the proportion of c-MET-overexpressing cancers between the HR+ /HER2− primary and mBC (*p* < 0.001).

### The prognostic impact of hotspot mutations and cfDNA concentration

**Fig. 3:**
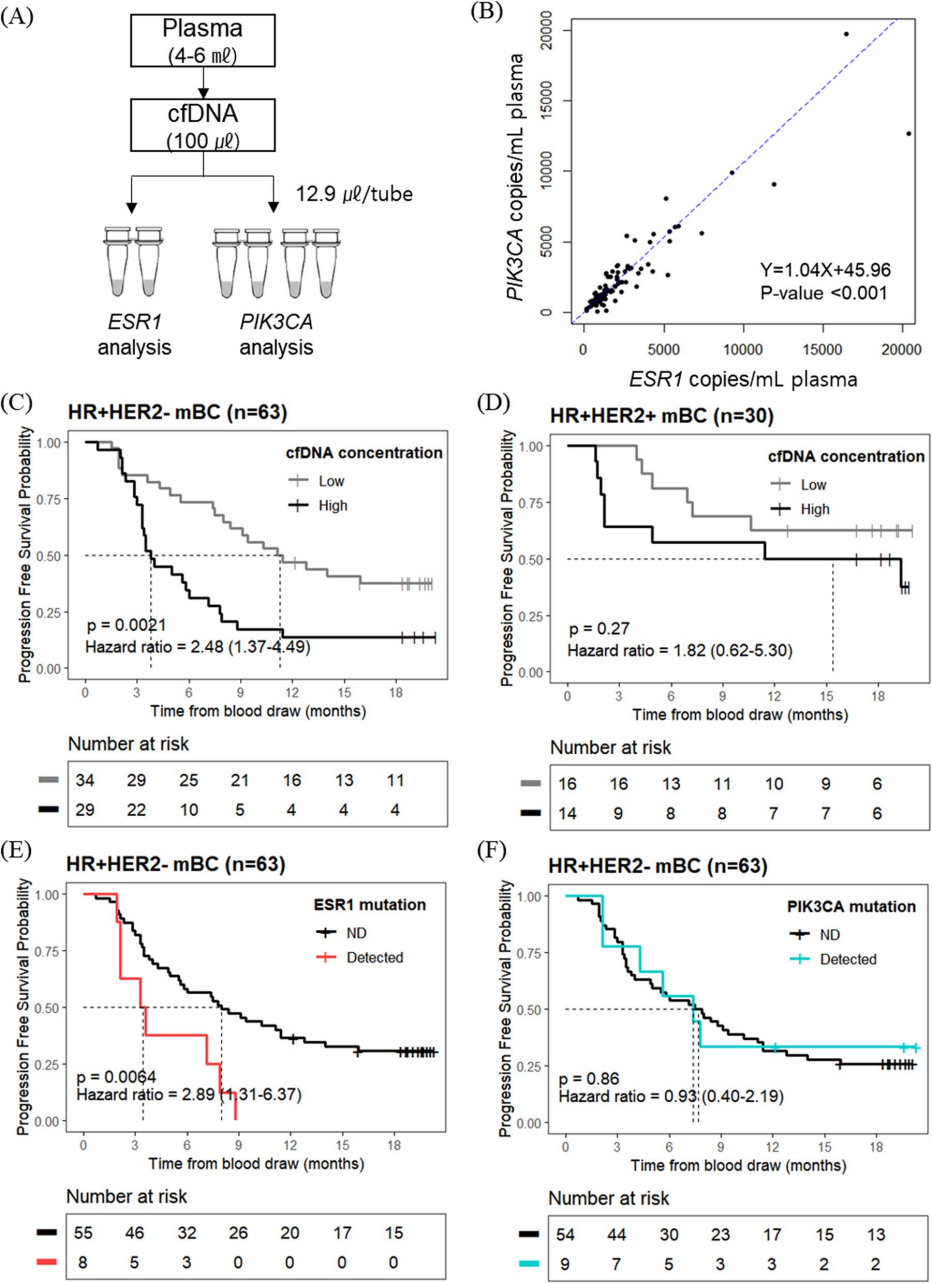
Progression impact of *ESR1* and *PIK3CA* concentration and mutations detected in cell-free DNA (cfDNA) samples. **A** Schematic diagram of the cfDNA analysis workflow. **B** Correlations between *ESR1* template copies/mL plasma and *PIK3CA* template copies/mL plasma. Kaplan–Meier analysis of PFS by **C** cfDNA concentration in HR+/HER2− mBC, **D** cfDNA concentration in HR+/HER2+ mBC, **E** *ESR1* hotspot mutation in HR+/HER2− mBC, and **F** *PIK3CA* hotspot mutation in HR+/HER2− mBC. *HR, hormone receptor; mBC, metastatic breast cancer; PFS, Progression-free survival*

The effects of cfDNA concentration and cfctDNA mutations on PFS in cfDNA samples were investigated, as shown in the cfDNA analysis scheme (Fig. 3A). Importantly, *ESR1* and *PIK3CA* copies/mL in plasma were strongly correlated (Pearson’s product-moment correlation coefficient = 0.92, *p* < 0.001; Fig. 3B).

The high cfDNA concentration group was significantly associated with reduced PFS in patients with HR+/HER2− mBC (*p* = 0.0021) (Fig. 3C), with a median PFS of 3.8 months (95% CI 3.3–7.1) in the high group compared to 11.2 months (95% CI 8.4–not estimable) in the low group. In patients with HR+/HER2+ BC, high cfDNA concentration was not associated with prognostic significance (*p* = 0.27) (Fig. 3D). Using the same cut-off of 1490 copies/mL of total *PIK3CA* templates in patients with HR+/HER2− mBC, the median PFS was 3.8 months (95% CI 3.3–7.9) in the high group compared to 12.1 months (95% CI 8.0–not estimable) in the low group (Additional file 8: Fig. S7).

CfctDNA Mutation analysis showed that 12.7% (8/63) of the patients with HR+/HER2− mBC had *ESR1* hotspot mutations, of which seven had a mutation in exon 8 (534–538). *ESR1* hotspot mutations resulted in reduced PFS (*p* = 0.0064) in these patients (Fig. 3E). In the *PIK3CA* hotspot mutation, 14.3% (9/63) of patients showed mutations, of which six had a mutation in exon 9 (542–546); however, the presence of *PIK3CA* mutation was not associated with poor prognosis (*p* = 0.86) in these patients (Fig. 3F).

### Univariate and multivariate analysis of PFS predictors

**Fig. 4:**
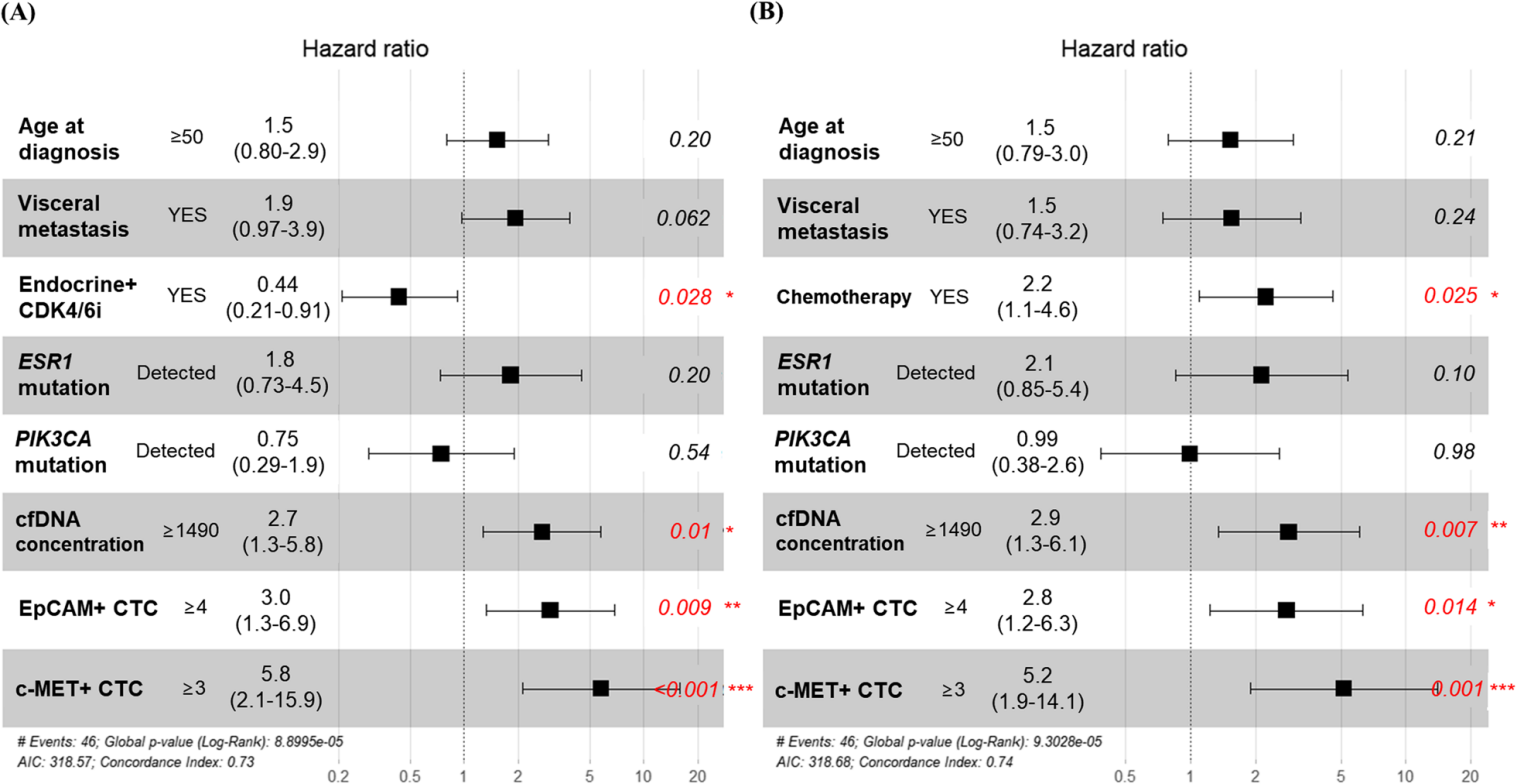
Forest plot. Cox regression multivariate analysis of the impact of various variables on progression-free survival in patients with HR+/HER2− mBC including **A** endocrine therapy combined with CDK4/6i (Endocrine + CDK4/6i) or **B** chemotherapy. *HR, hormone receptor; CTCs, circulating tumor cells; cfDNA, cell-free DNA*; *p < 0.05, **p < 0.01, ***p < 0.005

Univariate Cox proportional hazard regression analyses revealed an association between the patient characteristics of interest and PFS (Additional file 9: Table S8). In univariate analysis of the HR+/HER2− mBC, endocrine therapy combined with CDK4/6i (hazard ratio = 0.45, 95% CI 0.24–0.82, *p* = 0.0096), chemotherapy (hazard ratio = 2.2, 95% CI 1.2–4.0, *p* = 0.0074), cfDNA concentration (hazard ratio = 2.5, 95% CI 1.4–4.5, *p* = 0.0028), *ESR1* hotspot mutation (hazard ratio = 2.9, 1.3–6.4, *p* = 0.0084), and c-MET+ CTCs (hazard ratio = 3.6, 95% CI 1.5–9.0, *p* = 0.0047) were predictive factors for PFS. In contrast, the associations between PFS and EpCAM+ CTCs, c-MET+ CTCs, and cfDNA concentration were not significant in patients with HR+/HER2+ mBC. Multivariate analysis was conducted including endocrine therapy combined with CDK4/6i (Fig. 4A) or chemotherapy (Fig. 4B) along with other variables in HR+/HER2− mBC. Multivariate analysis including the use of endocrine therapy combined with CDK4/6i revealed that cfDNA concentration (hazard ratio = 2.7, 95% CI 1.3–5.8, *p* = 0.01), EpCAM+ CTCs (hazard ratio = 3.0, 95% CI 1.3–6.9, *p* = 0.009), and c-MET+ CTCs (hazard ratio = 5.8, 95% CI 2.1–15.9, *p* < 0.001) were independent predictors of progression in patients with HR+/HER2− mBC. In the case of chemotherapy, cfDNA concentration (HR = 2.9, 95% CI 1.3–6.1, *p* = 0.007), EpCAM+ CTCs (HR = 2.8, 95% CI 1.2–6.3, *p* = 0.014), and c-MET+ CTCs (HR = 5.2, 95% CI 1.9–14.1, *p* = 0.001) were also independent predictors of progression in patients with HR+/HER2− mBC. The baseline characteristics, CTC, cfDNA, and PFS data of each patient are presented in Additional file 10: Fig. S9.

### Survival analysis grouped by cfDNA concentration and c-MET+ CTCs or EpCAM+ CTCs

**Fig. 5:**
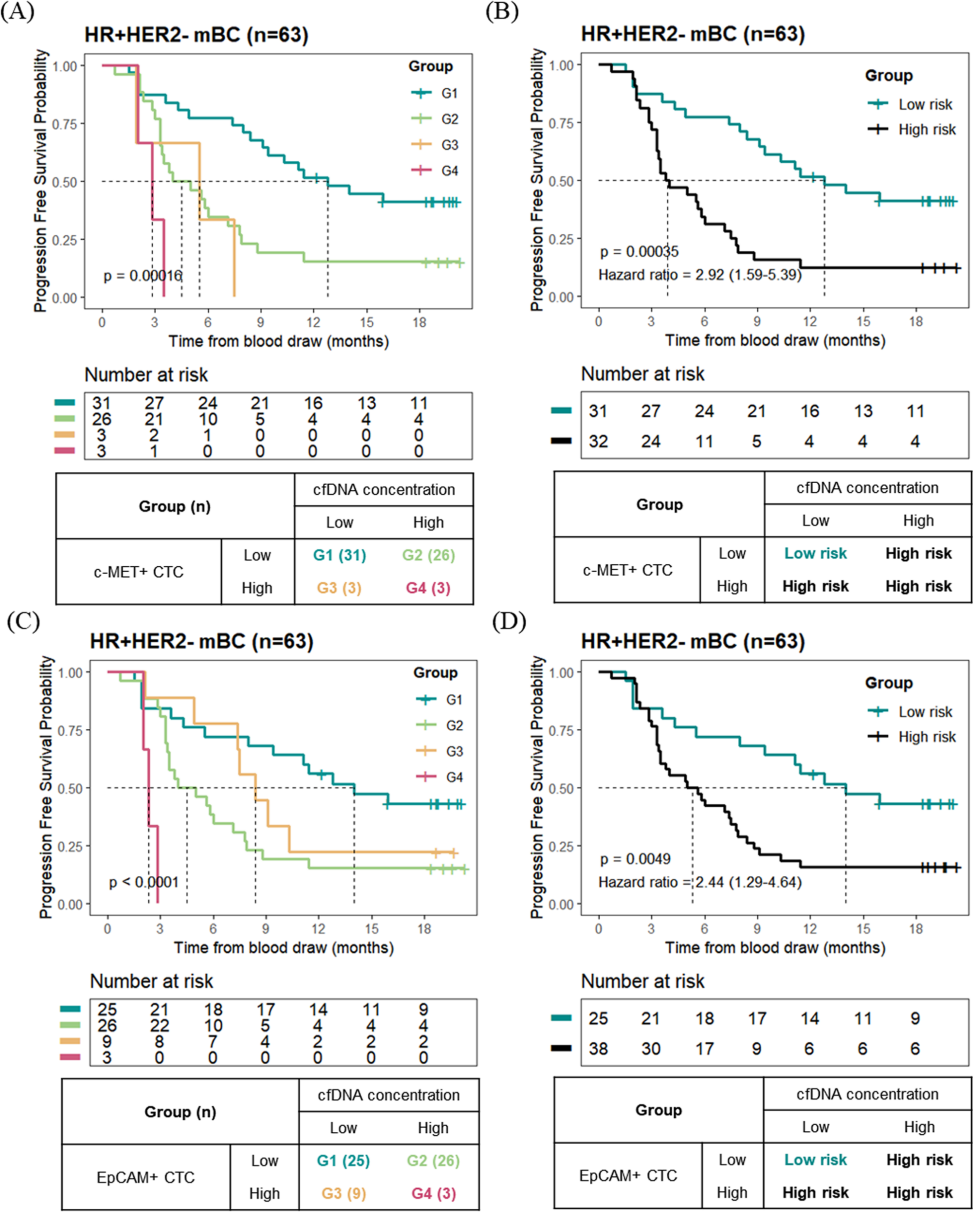
Survival analysis based on CTC and plasma cfDNA concentration in HR+/HER2− mBC. Kaplan–Meier analysis grouped by c-MET+ CTC (**A**, **B**) or EpCAM+ CTC (**C**, **D**) and cfDNA concentration. Patients with HR+/HER2− mBC were grouped into four (**A**, **C**) or two categories (**B**, **D**)

The combination of CTC- and cfDNA-derived information revealed their impact on the prognosis of patients with HR+/HER2− mBC. The patients were classified into four groups based on c-MET+ CTC count and cfDNA concentrations for Kaplan–Meier analysis. G1 included patients with low c-MET+ CTC counts and low cfDNA concentrations. Patients with low c-MET+ CTC counts and high cfDNA concentrations or high c-MET+ CTC counts and low cfDNA concentrations were classified into G2 and G3, respectively. G4 consisted of patients with high c-MET+ CTC and high cfDNA concentrations. Survival analysis among the four groups showed statistical significance (*p* < 0.001) in HR+/HER2− mBC patients (Fig. 5A). The median PFS of G1, G2, G3, and G4 was 12.8, 4.5, 5.5, and 2.8 months, respectively. Then, patients in G1 were re-classified into the low-risk group, and those in G2, G3, and G4 were classified as the high-risk group. The high-risk group comprised 50.8% (32/63) of patients and had a shorter PFS than the low-risk group (*p* < 0.001) (Fig. 5B). In high-risk and low-risk groups, median PFS was 3.9 months (95% CI 3.3–7.1) and 12.8 months (95% CI 9.1–not estimable), respectively. The PFS at 6 months in the low-risk and high-risk groups classified according to c-MET+ CTC count and cfDNA concentration was 77.4% (95% CI 64.0–93.6) and 31.2% (95% CI 18.7–52.2), respectively.

We evaluated the prognostic value of EpCAM+ CTCs and cfDNA concentrations using the same approach. The patients were grouped based on their EpCAM+ CTC and cfDNA concentrations, and Kaplan–Meier analysis revealed statistical significance (*p* < 0.001) among the four groups (Fig. 5C). After the patients were classified into low-risk or high-risk groups, 60.3% (38/63) of the patients were classified into the high-risk group, with significantly shorter PFS than the low-risk group (*p* = 0.0049) (Fig. 5D). In the high-risk and low-risk groups, the median PFS was 5.3 months (95% CI 3.5–7.9) and 14.0 months (95% CI 9.4–not estimable), respectively. The PFS at 6 months in the low-risk and high-risk groups categorized by EpCAM+ CTC count and cfDNA concentration was 72.0% (95% CI 56.4–91.9) and 42.1% (95% CI 29.0–61.1), respectively.

## Discussion

The primary objective of the present study was to investigate the prognostic value of c-MET+ CTC or EpCAM+ CTC analysis, cfctDNA-derived *ESR1* and *PIK3CA* mutations, and cfDNA concentration in patients with HR+ mBC. This is the first study to evaluate the integrated prognostic value of c-MET+ CTCs and cfDNA concentrations in patients with HR+ mBC. Although several studies have shown EpCAM+ CTCs as prognostic biomarkers in different cancer types, very few studies have evaluated c-MET expression in CTCs. Several studies have reported that c-MET expression in CTCs or c-MET+ CTCs can be detected in patients with cancer [30, 31]; however, survival analysis has not been conducted. One study reported that c-MET expression in CTCs enriched by size-based filtration showed poorer OS in a small number of patients with head and neck cancers (*n* = 11) but not in patients with BC [32].

The CellSearch^®^ system showed that EpCAM+ CTCs were correlated with poor prognosis in cancer patients [33]. However, several studies have suggested that the count of CTCs with EMT phenotypes may be more appropriate than that of the epithelial phenotype for predicting therapeutic resistance and assessing prognosis [9, 10, 34]. Because c-MET signaling is associated with EMT [35, 36] and therapeutic resistance [22, 25], c-MET+ CTCs can provide information for predicting disease progression or therapeutic resistance. Since c-MET-overexpressing tumors in patients with HR+ mBC have shorter PFS and a higher frequency of visceral metastases [22, 37], c-MET+ CTCs are expected to be associated with disease progression.

In the present study, we detected c-MET+ CTCs in patients with HR+ mBC. Although 70% of patients with mBC are reported to have one or more CTCs/7.5 mL blood [38], only 46.2% (43/93) of patients with HR+ mBC had one or more EpCAM+ or c-MET+ CTCs/4 mL blood in the present study. This could be attributed to the small blood volume utilized for CTC enrichment (4 mL) or differences in the methods used for CTC enrichment. There was a significant difference in the number of c-MET+ CTCs between patients with visceral metastasis, especially in liver metastasis, compared to those with non-visceral metastasis who had detectable CTCs in their blood. However, given the limited sample size, caution is advised in interpreting the data.

In survival analysis, c-MET+ CTCs were associated with reduced PFS (*p* = 0.0026) in the HR+/HER2− mBC patients. In contrast, although several previous studies have shown that EpCAM+ CTCs are associated with prognosis [6], EpCAM+ CTCs in our cohort had no prognostic significance for PFS (*p* = 0.38). These results suggested that c-MET+ CTCs may have a more substantial prognostic impact than EpCAM+ CTCs in HR+/HER2− mBC. Given that the combined analysis of EpCAM+ CTCs and cfDNA concentration showed a significant association with PFS (*p* = 0.0049), patients with high cfDNA concentrations in the EpCAM+ CTCs low group might have decreased the prognostic value of EpCAM+ CTCs in our cohort. In addition, forest plot analysis showed that EpCAM+ CTCs had prognostic significance for PFS, suggesting that other factors affected the prognostic value of EpCAM+ CTCs in our cohort. We did not observe a significant correlation between c-MET+ CTCs or cfDNA concentration and PFS in HR+/HER2+ patients. Although this cannot be proven, we believe that it is mainly due to the small sample size of patients with HR+/HER2+ mBC (*n* = 30).

To investigate the need to monitor c-MET+ CTCs in the blood, we evaluated the differences in c-MET overexpression rates between the primary and metastatic sites of HR+/HER2− BC. In the primary BC cohort (SMC-BCBS TMA), c-MET overexpression in HR+/HER2−, HR+/HER2+, HER2-enriched, and TNBC cases was 4.7%, 3.8%, 7.1%, and 13.6%, respectively. These results are similar to those of previous studies [39, 40]. Although HR+/HER2− BC metastatic sites were independently collected, 22.2% were classified as having c-MET overexpression. Moreover, the proportion of c-MET-overexpressing cancers was significantly higher in metastatic sites than in primary BC (*p* = 0.00002) in HR+/HER2− BC. The proportion of c-MET overexpression increases at metastatic sites; hence, monitoring c-MET-expressing cells rather than examining c-MET expression in the primary breast could provide valuable information for predicting the prognosis of HR+/HER2− mBC.

Based on the responsiveness of highly selective c-MET inhibitors to c-MET overexpressing cancer, we believe that the reduced PFS in the c-MET+ CTC high group suggests the potential effectiveness of c-MET inhibitors in treating patients with HR+/HER2− mBC. Indeed, treatment modalities for patients with metastatic non-small cell lung cancer have changed since the US FDA approved c-MET inhibitors such as capmatinib (Tabrecta^®^) and tepotinib (Tepmetko^®^). Moreover, several clinical trials have reported the effectiveness of tepotinib and teliso-V in cancers overexpressing c-MET [41–43]. Although these clinical trials did not include patients with BC, c-MET inhibitors might improve the outcome of patients with HR+/HER2− with high c-MET+ CTCs, indicating the presence of cancer cells expressing c-MET. Further clinical trials are needed to evaluate the cut-off values of c-MET+ CTCs for the treatment of HR+/HER2− mBC with c-MET inhibitors.

*ESR1* and *PIK3CA* alterations are frequently observed in patients with mBC and have been identified in cfctDNA samples [44]. We used the ddPCR platform to detect *ESR1* and *PIK3CA* hotspot mutations and calculate cfDNA concentrations, providing absolute quantification of nucleic acids without quantifying DNA concentrations using other techniques [45]. *ESR1* and *PIK3CA* ctDNA mutations were detected in 12.7% and 14.3% of patients with HR+ mBC in cfDNA samples, respectively, and only *ESR1* mutations were associated with shorter PFS (log-rank, *p* = 0.0064). The high correlation between *ESR1* and *PIK3CA* template copy numbers suggests that cfDNA evenly represents DNA fragments from the cell. Consistent with previous studies [46, 47], cfDNA concentration was associated with prognosis in patients with HR+/HER2− mBC (log-rank test, *p* = 0.0021).

According to multivariate Cox regression analysis, c-MET+ CTCs, EpCAM+ CTCs, and cfDNA concentrations were independent predictors of disease progression in patients with HR+/HER2− mBC. The current study demonstrated that CTCs and cfDNA can provide complementary information regarding disease progression, emphasizing the importance of integrated liquid biopsy. These findings are consistent with those of previous studies [48–50].

This study had several limitations. First, patients were not enrolled at the time of treatment initiation, even though the intervals between treatment initiation and blood draw were not significant when grouped by cMET+ CTC, EpCAM+ CTC, or cfDNA concentration in HR+/HER2− or HR+/HER2+ mBC. Further studies are required to determine the optimal monitoring intervals of each LBx analyte for achieving the best cost-effectiveness. Secondly, the cut-off values of each biomarker were calculated retrospectively, which may have led to overfitting. Appropriate cut-off values need to be confirmed in further studies of independent cohorts.

Regardless of these limitations, this study highlights c-MET+ CTCs and cfDNA concentrations as significant independent predictors of progression in patients with HR+/HER2− mBC. Further prospective studies are required to validate each biomarker, including the cut-off values and optimal time for testing. Based on the results of this study, a prospective clinical trial to evaluate the combination of ABN401, a highly selective MET inhibitor [51], with standard-of-care is planned in patients with HR+/HER2− mBC who have c-MET+ CTC, which could show predictive value of c-MET+ CTC.

## Supplementary Information

**Additional file 1.** Supplementary Methods.

**Additional file 2.** **Supplementary Fig. S1**. The interval between treatment initiation and blood draw grouped by (A) cMET+ CTC, (B) EpCAM+ CTC, and (C) cfDNA concentration. HR, hormone receptor; CTC, circulating tumor cell; cfDNA, cell-free DNA; SD, standard deviation.

**Additional file 3.** **Supplementary Fig. S2**. Analytical performance of the c-MET+ CTC assay used in the study. (A) Flow cytometry analysis of SNU5 and MCF7 cells labeled with 2 μg/mL of anti c-MET antibodies. (B) Recovery rates and separation rates of SNU5 and MCF7 cells spiked into buffer (left) and normal human blood (right). Data showed average +/- standard deviation from three experiments. (C) Total number of white blood cells (WBCs) contaminated during CTC enrichment per 4 mL of patient blood. CTC, circulating tumor cells.

**Additional file 4.** **Supplementary Fig. S3**. The number of c-MET+ CTCs in patients with detected CTCs by the site of metastasis. Others refers to cases where the visceral metastatic site was other than the liver.

**Additional file 5.** **Supplementary Table S4.** Baseline characteristics of primary and metastatic sites in HR+/HER2- breast cancer.

**Additional file 6.** **Supplementary Fig. S5**. c-MET overexpression in primary breast and metastatic sites in patients with HR+HER2- mBC. (A) Representative immunohistochemical staining intensities for c-MET expression and (B) proportion of c-MET overexpression (positive) in primary breast and metastatic sites. Tumor samples of primary breast and metastatic sites were independently collected and scored.mBC, metastatic breast cancer.

**Additional file 7.** **Supplementary Table S6**. Summary of previous and present studies on positive rate of c-MET overexpression in breast cancer.

**Additional file 8.** **Supplementary Fig. S7**. Survival analysis using cell-free DNA concentration calculated from the internal control copies of the Droplex PIK3CA Mutation Test Kit, with a cut-off value of 1490 copies/mL plasma in (A) HR+HER2- mBC or (B) HR+HER2+ mBC. mBC, metastatic breast cancer; cfDNA, cell-free DNA.

**Additional file 9.** **Supplementary Table S8**. Univariate Cox proportional hazard models for PFS.

**Additional file 10.** **Supplementary Fig. S9**. Baseline characteristics, CTC, cfDNA, and PFS data of each patient Tx, Treatment; CTC, circulating tumor cell; Conc., concentration; PFS, progression-free survival.

## Data Availability

The datasets used and/or analyzed during the current study are available from the corresponding author on reasonable request.

## References

[1] Haque MM, Desai KV. Pathways to endocrine therapy resistance in breast cancer. Front Endocrinol. 2019. 10.3389/fendo.2019.00573. 10.3389/fendo.2019.00573 PMC6712962 31496995

[2] Xu X-Q, Pan X-H, Wang T-T, Wang J, Yang B, He Q-J, et al. Intrinsic and acquired resistance to CDK4/6 inhibitors and potential overcoming strategies. Acta Pharmacol Sin. 2021;42(2):171–8. 10.1038/s41401-020-0416-4. 32504067 10.1038/s41401-020-0416-4 PMC8027849

[3] Hanker AB, Sudhan DR, Arteaga CL. Overcoming endocrine resistance in breast cancer. Cancer Cell. 2020;37(4):496–513. 10.1016/j.ccell.2020.03.009. 32289273 10.1016/j.ccell.2020.03.009 PMC7169993

[4] Pantel K, Alix-Panabieres C. Liquid biopsy and minimal residual disease—latest advances and implications for cure. Nat Rev Clin Oncol. 2019;16(7):409–24. 10.1038/s41571-019-0187-3. 30796368 10.1038/s41571-019-0187-3

[5] Cristofanilli M, Budd GT, Ellis MJ, Stopeck A, Matera J, Miller MC, et al. Circulating tumor cells, disease progression, and survival in metastatic breast cancer. N Engl J Med. 2004;351(8):781–91. 10.1056/NEJMoa040766. 15317891 10.1056/NEJMoa040766

[6] Hayes DF, Cristofanilli M, Budd GT, Ellis MJ, Stopeck A, Miller MC, et al. Circulating tumor cells at each follow-up time point during therapy of metastatic breast cancer patients predict progression-free and overall survival. Clin Cancer Res. 2006;12(14 Pt 1):4218–24. 10.1158/1078-0432.CCR-05-2821. 16857794 10.1158/1078-0432.CCR-05-2821

[7] Nole F, Munzone E, Zorzino L, Minchella I, Salvatici M, Botteri E, et al. Variation of circulating tumor cell levels during treatment of metastatic breast cancer: prognostic and therapeutic implications. Ann Oncol. 2008;19(5):891–7. 10.1093/annonc/mdm558. 18056915 10.1093/annonc/mdm558

[8] Gorges TM, Tinhofer I, Drosch M, Röse L, Zollner TM, Krahn T, et al. Circulating tumour cells escape from EpCAM-based detection due to epithelial-to-mesenchymal transition. BMC Cancer. 2012;12(1):178. 10.1186/1471-2407-12-178. 22591372 10.1186/1471-2407-12-178 PMC3502112

[9] Horimoto Y, Tokuda E, Murakami F, Uomori T, Himuro T, Nakai K, et al. Analysis of circulating tumour cell and the epithelial mesenchymal transition (EMT) status during eribulin-based treatment in 22 patients with metastatic breast cancer: a pilot study. J Transl Med. 2018;16(1):287. 10.1186/s12967-018-1663-8. 30342534 10.1186/s12967-018-1663-8 PMC6195982

[10] Yu M, Bardia A, Wittner BS, Stott SL, Smas ME, Ting DT, et al. Circulating breast tumor cells exhibit dynamic changes in epithelial and mesenchymal composition. Science (New York, NY). 2013;339(6119):580–4. 10.1126/science.1228522. 10.1126/science.1228522 PMC3760262 23372014

[11] Cohen EN, Jayachandran G, Moore RG, Cristofanilli M, Lang JE, Khoury JD, et al. A multi-center clinical study to harvest and characterize circulating tumor cells from patients with metastatic breast cancer using the Parsortix^®^ PC1 system. Cancers. 2022;14(21):5238. 36358657 10.3390/cancers14215238 PMC9656921

[12] Page K, Guttery DS, Fernandez-Garcia D, Hills A, Hastings RK, Luo J, et al. Next generation sequencing of circulating cell-free DNA for evaluating mutations and gene amplification in metastatic breast cancer. Clin Chem. 2017;63(2):532–41. 10.1373/clinchem.2016.261834. 27940449 10.1373/clinchem.2016.261834 PMC6241835

[13] Takeshita T, Yamamoto Y, Yamamoto-Ibusuki M, Inao T, Sueta A, Fujiwara S, et al. Droplet digital polymerase chain reaction assay for screening of ESR1 mutations in 325 breast cancer specimens. Transl Res. 2015;166(6):540–53. 10.1016/j.trsl.2015.09.003. 26434753 10.1016/j.trsl.2015.09.003

[14] Jeselsohn R, Yelensky R, Buchwalter G, Frampton G, Meric-Bernstam F, Gonzalez-Angulo AM, et al. Emergence of constitutively active estrogen receptor-alpha mutations in pretreated advanced estrogen receptor-positive breast cancer. Clin Cancer Res. 2014;20(7):1757–67. 10.1158/1078-0432.CCR-13-2332. 24398047 10.1158/1078-0432.CCR-13-2332 PMC3998833

[15] Fribbens C, O’Leary B, Kilburn L, Hrebien S, Garcia-Murillas I, Beaney M, et al. Plasma ESR1 mutations and the treatment of estrogen receptor-positive advanced breast cancer. J Clin Oncol. 2016;34(25):2961–8. 10.1200/jco.2016.67.3061. 27269946 10.1200/JCO.2016.67.3061

[16] Cristofanilli M, Turner NC, Bondarenko I, Ro J, Im SA, Masuda N, et al. Fulvestrant plus palbociclib versus fulvestrant plus placebo for treatment of hormone-receptor-positive, HER2-negative metastatic breast cancer that progressed on previous endocrine therapy (PALOMA-3): final analysis of the multicentre, double-blind, phase 3 randomised controlled trial. Lancet Oncol. 2016;17(4):425–39. 10.1016/S1470-2045(15)00613-0. 26947331 10.1016/S1470-2045(15)00613-0

[17] Wu JM, Fackler MJ, Halushka MK, Molavi DW, Taylor ME, Teo WW, et al. Heterogeneity of breast cancer metastases: comparison of therapeutic target expression and promoter methylation between primary tumors and their multifocal metastases. Clin Cancer Res. 2008;14(7):1938–46. 10.1158/1078-0432.CCR-07-4082. 18381931 10.1158/1078-0432.CCR-07-4082 PMC2965068

[18] Van Poznak C, Somerfield MR, Bast RC, Cristofanilli M, Goetz MP, Gonzalez-Angulo AM, et al. Use of biomarkers to guide decisions on systemic therapy for women with metastatic breast cancer: American Society of Clinical Oncology Clinical Practice Guideline. J Clin Oncol. 2015;33(24):2695–704. 10.1200/JCO.2015.61.1459. 26195705 10.1200/JCO.2015.61.1459 PMC5478102

[19] Burstein HJ, DeMichele A, Somerfield MR, Henry NL. Testing for ESR1 mutations to guide therapy for hormone receptor-positive, human epidermal growth factor receptor 2–negative metastatic breast cancer: ASCO Guideline Rapid Recommendation Update. J Clin Oncol. 2023. 10.1200/jco.23.00638. 37196213 10.1200/JCO.23.00638

[20] Weng T-H, Yao M-Y, Xu X-M, Hu C-Y, Yao S-H, Liu Y-Z, et al. RON and MET co-overexpression are significant pathological characteristics of poor survival and therapeutic targets of tyrosine kinase inhibitors in triple-negative breast cancer. Cancer Res Treat. 2020;52(3):973–86. 10.4143/crt.2019.726. 32324988 10.4143/crt.2019.726 PMC7373856

[21] Fu J, Su X, Li Z, Deng L, Liu X, Feng X, et al. HGF/c-MET pathway in cancer: from molecular characterization to clinical evidence. Oncogene. 2021;40(28):4625–51. 10.1038/s41388-021-01863-w. 34145400 10.1038/s41388-021-01863-w

[22] Van den Bossche V, Jadot G, Grisay G, Pierrard J, Honoré N, Petit B, et al. c-MET as a potential resistance mechanism to everolimus in breast cancer: from a case report to patient cohort analysis. Target Oncol. 2020;15(1):139–46. 10.1007/s11523-020-00704-2. 32020516 10.1007/s11523-020-00704-2

[23] Goetz MP, Hamilton EP, Campone M, Hurvitz SA, Cortes J, Johnston SRD, et al. Acquired genomic alterations in circulating tumor DNA from patients receiving abemaciclib alone or in combination with endocrine therapy. J Clin Oncol. 2020;38(15_suppl):3519. 10.1200/JCO.2020.38.15_suppl.3519.

[24] Liao H, Tian T, Sheng Y, Peng Z, Li Z, Wang J, et al. The significance of MET expression and strategies of targeting MET treatment in advanced gastric cancer. Front Oncol. 2021. 10.3389/fonc.2021.719217. 34557411 10.3389/fonc.2021.719217 PMC8453156

[25] Wood GE, Hockings H, Hilton DM, Kermorgant S. The role of MET in chemotherapy resistance. Oncogene. 2021;40(11):1927–41. 10.1038/s41388-020-01577-5. 33526881 10.1038/s41388-020-01577-5 PMC7979538

[26] Eisenhauer EA, Therasse P, Bogaerts J, Schwartz LH, Sargent D, Ford R, et al. New response evaluation criteria in solid tumours: revised RECIST guideline (version 1.1). Eur J Cancer. 2009;45(2):228–47. 10.1016/j.ejca.2008.10.026. 19097774 10.1016/j.ejca.2008.10.026

[27] Chelakkot C, Ryu J, Kim MY, Kim JS, Kim D, Hwang J, et al. An immune-magnetophoretic device for the selective and precise enrichment of circulating tumor cells from whole blood. Micromachines (Basel). 2020. 10.3390/mi11060560. 32486306 10.3390/mi11060560 PMC7345362

[28] Choi Y-L, Oh E, Park S, Kim Y, Park Y-H, Song K, et al. Triple-negative, basal-like, and quintuple-negative breast cancers: better prediction model for survival. BMC Cancer. 2010;10(1):507. 10.1186/1471-2407-10-507. 20860845 10.1186/1471-2407-10-507 PMC2957395

[29] Kim YJ, Choi J-S, Seo J, Song J-Y, Eun Lee S, Kwon MJ, et al. MET is a potential target for use in combination therapy with EGFR inhibition in triple-negative/basal-like breast cancer. Int J Cancer. 2014;134(10):2424–36. 10.1002/ijc.28566. 24615768 10.1002/ijc.28566

[30] Zhang T, Boominathan R, Foulk B, Rao C, Kemeny G, Strickler JH, et al. Development of a novel c-MET-based CTC detection platform. Mol Cancer Res. 2016;14(6):539–47. 10.1158/1541-7786.MCR-16-0011. 26951228 10.1158/1541-7786.MCR-16-0011

[31] Ilie M, Szafer-Glusman E, Hofman V, Long-Mira E, Suttmann R, Darbonne W, et al. Expression of MET in circulating tumor cells correlates with expression in tumor tissue from advanced-stage lung cancer patients. Oncotarget. 2017;8(16):26112–21. 10.18632/oncotarget.15345. 10.18632/oncotarget.15345 PMC5432243 28212540

[32] Mondelo-Macía P, Rodríguez-López C, Valiña L, Aguín S, León-Mateos L, García-González J, et al. Detection of MET alterations using cell free DNA and circulating tumor cells from cancer patients. Cells. 2020;9(2):522. 32102486 10.3390/cells9020522 PMC7072825

[33] Bidard FC, Peeters DJ, Fehm T, Nolé F, Gisbert-Criado R, Mavroudis D, et al. Clinical validity of circulating tumour cells in patients with metastatic breast cancer: a pooled analysis of individual patient data. Lancet Oncol. 2014;15(4):406–14. 10.1016/s1470-2045(14)70069-5. 24636208 10.1016/S1470-2045(14)70069-5

[34] Mego M, Karaba M, Minarik G, Benca J, Silvia J, Sedlackova T, et al. Circulating tumor cells with epithelial–to–mesenchymal transition phenotypes associated with inferior outcomes in primary breast cancer. Anticancer Res. 2019;39(4):1829–37. 10.21873/anticanres.13290. 10.21873/anticanres.13290 30952723

[35] Wang S, Ma H, Yan Y, Chen Y, Fu S, Wang J, et al. cMET promotes metastasis and epithelial-mesenchymal transition in colorectal carcinoma by repressing RKIP. J Cell Physiol. 2021;236(5):3963–78. 10.1002/jcp.30142. 33151569 10.1002/jcp.30142

[36] Jeon HM, Lee J. MET: roles in epithelial-mesenchymal transition and cancer stemness. Ann Transl Med. 2017;5(1):5. 10.21037/atm.2016.12.67. 28164090 10.21037/atm.2016.12.67 PMC5253283

[37] Raghav KP, Wang W, Liu S, Chavez-MacGregor M, Meng X, Hortobagyi GN, et al. cMET and phospho-cMET protein levels in breast cancers and survival outcomes. Clin Cancer Res. 2012;18(8):2269–77. 10.1158/1078-0432.Ccr-11-2830. 22374333 10.1158/1078-0432.CCR-11-2830 PMC3821167

[38] Miller MC, Doyle GV, Terstappen LWMM. Significance of circulating tumor cells detected by the cell search system in patients with metastatic breast colorectal and prostate cancer. J Oncol. 2010;2010:617421. 10.1155/2010/617421. 20016752 10.1155/2010/617421 PMC2793426

[39] Wang M, Liang L, Lei X, Multani A, Meric-Bernstam F, Tripathy D, et al. Evaluation of cMET aberration by immunohistochemistry and fluorescence in situ hybridization (FISH) in triple negative breast cancers. Ann Diagn Pathol. 2018;35:69–76. 10.1016/j.anndiagpath.2018.04.004. 29843069 10.1016/j.anndiagpath.2018.04.004

[40] Zagouri F, Brandstetter A, Moussiolis D, Chrysikos D, Dimitrakakis C, Tsigginou A, et al. Low protein expression of MET in ER-positive and HER2-positive breast cancer. Anticancer Res. 2014;34(3):1227–31. 24596364

[41] Camidge DR, Moiseenko F, Cicin I, Horinouchi H, Filippova E, Bar J, et al. Abstract CT179: telisotuzumab vedotin (teliso-v) monotherapy in patients with previously treated c-Met+ advanced non-small cell lung cancer. Cancer Res. 2021;81(13_Supplement):CT179-CT. 10.1158/1538-7445.Am2021-ct179.

[42] Falchook GS, Kurzrock R, Amin HM, Xiong W, Fu S, Piha-Paul SA, et al. First-in-man phase I trial of the selective MET inhibitor tepotinib in patients with advanced solid tumors. Clin Cancer Res. 2020;26(6):1237–46. 10.1158/1078-0432.Ccr-19-2860. 31822497 10.1158/1078-0432.CCR-19-2860

[43] Wu YL, Cheng Y, Zhou J, Lu S, Zhang Y, Zhao J, et al. Tepotinib plus gefitinib in patients with EGFR-mutant non-small-cell lung cancer with MET overexpression or MET amplification and acquired resistance to previous EGFR inhibitor (INSIGHT study): an open-label, phase 1b/2, multicentre, randomised trial. Lancet Respir Med. 2020;8(11):1132–43. 10.1016/s2213-2600(20)30154-5. 32479794 10.1016/S2213-2600(20)30154-5

[44] Davis AA, Jacob S, Gerratana L, Shah AN, Wehbe F, Katam N, et al. Landscape of circulating tumour DNA in metastatic breast cancer. EBioMedicine. 2020;58:102914. 10.1016/j.ebiom.2020.102914. 32707446 10.1016/j.ebiom.2020.102914 PMC7381501

[45] Hindson BJ, Ness KD, Masquelier DA, Belgrader P, Heredia NJ, Makarewicz AJ, et al. High-throughput droplet digital PCR system for absolute quantitation of DNA copy number. Anal Chem. 2011;83(22):8604–10. 10.1021/ac202028g. 22035192 10.1021/ac202028g PMC3216358

[46] Cheng J, Holland-Letz T, Wallwiener M, Surowy H, Cuk K, Schott S, et al. Circulating free DNA integrity and concentration as independent prognostic markers in metastatic breast cancer. Breast Cancer Res Treat. 2018;169(1):69–82. 10.1007/s10549-018-4666-5. 29340881 10.1007/s10549-018-4666-5

[47] Fernandez-Garcia D, Hills A, Page K, Hastings RK, Toghill B, Goddard KS, et al. Plasma cell-free DNA (cfDNA) as a predictive and prognostic marker in patients with metastatic breast cancer. Breast Cancer Res. 2019;21(1):149. 10.1186/s13058-019-1235-8. 31856868 10.1186/s13058-019-1235-8 PMC6924016

[48] Bortolini Silveira A, Bidard F-C, Tanguy M-L, Girard E, Trédan O, Dubot C, et al. Multimodal liquid biopsy for early monitoring and outcome prediction of chemotherapy in metastatic breast cancer. npj Breast Cancer. 2021;7(1):115. 10.1038/s41523-021-00319-4. 34504096 10.1038/s41523-021-00319-4 PMC8429692

[49] Keup C, Suryaprakash V, Hauch S, Storbeck M, Hahn P, Sprenger-Haussels M, et al. Integrative statistical analyses of multiple liquid biopsy analytes in metastatic breast cancer. Genome Med. 2021;13(1):85. 10.1186/s13073-021-00902-1. 34001236 10.1186/s13073-021-00902-1 PMC8130163

[50] Ye Z, Wang C, Wan S, Mu Z, Zhang Z, Abu-Khalaf MM, et al. Association of clinical outcomes in metastatic breast cancer patients with circulating tumour cell and circulating cell-free DNA. Eur J Cancer. 2019;106:133–43. 10.1016/j.ejca.2018.10.012. 30528798 10.1016/j.ejca.2018.10.012 PMC6347110

[51] Kim J, Park KE, Jeong YS, Kim Y, Park H, Nam JH, et al. Therapeutic efficacy of ABN401, a highly potent and selective MET inhibitor, based on diagnostic biomarker test in MET-addicted cancer. Cancers (Basel). 2020. 10.3390/cancers12061575. 32549194 10.3390/cancers12061575 PMC7352216

