# Correction: c-MET-positive circulating tumor cells and cell-free DNA as independent prognostic factors in hormone receptor-positive/HER2-negative metastatic breast cancer

**DOI:** 10.1186/s13058-024-01952-0

**Published:** 2025-01-07

**Authors:** Jieun Park, Eun Sol Chang, Ji-Yeon Kim, Chaithanya Chelakkot, Minjung Sung, Ji-Young Song, Kyungsoo Jung, Ji Hye Lee, Jun Young Choi, Na Young Kim, Hyegyeong Lee, Mi-Ran Kang, Mi Jeong Kwon, Young Kee Shin, Yeon Hee Park, Yoon-La Choi

**Affiliations:** 1 Department of Molecular Medicine and Biopharmaceutical Sciences, Graduate School of Convergence Science and Technology, Seoul National University, Seoul, 08826 Republic of Korea; 2 Department of Health Sciences and Technology, SAIHST, Sungkyunkwan University, Seoul, Republic of Korea; 3 Laboratory of Molecular Pathology and Theranostics, Samsung Medical Center, Sungkyunkwan University School of Medicine, Seoul, Republic of Korea; 4 Division of Hematology-Oncology, Department of Medicine, Samsung Medical Center, Sungkyunkwan University School of Medicine, Irwon-ro 81, Gangnam-gu, Seoul, 06351 Republic of Korea; 5 Technical Research Center, Genobio Corp., Seoul, Republic of Korea; 6 Laboratory of Molecular Pathology and Cancer Genomics, College of Pharmacy and Research Institute of Pharmaceutical Sciences, Seoul National University, Seoul, Republic of Korea; 7 Department of Pathology and Translational Genomics, Samsung Medical Center, Sungkyunkwan University School of Medicine, Irwon-ro 81, Gangnam-gu, Seoul, 06351 Republic of Korea; 8 R&D Center, ABION Inc., Seoul, Republic of Korea; 9 Central Laboratory, LOGONE Bio-Convergence Research Foundation, Seoul, Republic of Korea; 10 R&D Center, Gencurix Inc., Seoul, Republic of Korea; 11 Vessel-Organ Interaction Research Center (MRC), College of Pharmacy, Kyungpook National University, Daegu, Republic of Korea; 12 BK21 FOUR Community-Based Intelligent Novel Drug Discovery Education Unit, College of Pharmacy and Research Institute of Pharmaceutical Sciences, Kyungpook National University, Daegu, Republic of Korea

**Correction to: Park et al. Breast Cancer Research (2024) 26:13**

10.1186/s13058-024-01768-y

Following the publication of the original article, the authors reported that an abbreviation in the Background section of the manuscript, specifically, in the fourth paragraph, was inaccurate.

**Incorect**

Mesenchymal-epithelial transition factor (MET).

**Correct**

MET (MNNG-HOS transforming gene).

The Original Article has been corrected.

