# Supplementary material for: c-MET-positive circulating tumor cells and cell-free DNA as independent prognostic factors in hormone receptor-positive/HER2-negative metastatic breast cancer
Source: Breast Cancer Res. 2024 Jan 18;26:13. doi: 10.1186/s13058-024-01768-y (PMC10797795; doi:10.1186/s13058-024-01768-y)
Supplement: Supplementary file 1 — Additional file 1. Supplementary Methods. [file 13058_2024_1768_MOESM1_ESM.docx]

Supplementary Methods

Flow cytometry

To confirm the expression levels of cell surface c-MET in the cell lines, flow cytometry (FACS) analysis was performed. SNU5 or MCF7 cells (2×10^5^ cells) were incubated with 2 µg/mL biotinylated anti-human c-MET antibody included in the c-MET isolation kit (Genobio Corp., Seoul, Republic of Korea) at 4ºC for 1 h. After incubation, cells were washed once with PBS supplemented with 1% FBS. Subsequently, 1 µg/mL FITC-conjugated streptavidin (Jackson ImmunoResearch, West Grove, PA, USA; Cat#016-540-084) was incubated for 1 h at 4ºC in the dark. Cells were washed thrice, and cell surface expression of c-MET was analyzed using a BD FACSCalibur cytometer (BD Bioscience, MA, USA). The results were analyzed using Flowing Software version 2.5.1 (University of Turku, Finland).

Spiked cell enrichment

A c-MET isolation kit (Genobio Corp) was used for the spiking experiments. Final 1–3 or 10×10^3^ cells were spiked into 1 mL culture medium or healthy human blood and incubated with anti-human c-MET antibodies conjugated with magnetic beads for 30–60 min at room temperature, as described by the manufacturer. After incubation, samples were loaded into GenoCTC® for cell isolation. For the healthy human blood spiking test, cells were stained with green fluorescence using Cell Tracking Dye (Abcam, Cambridge, MA, USA; Cat#ab138891) before spiking the blood to avoid confusion with white blood cell contamination.

Spiked cell enumeration

Isolated cells and samples collected from the waste line were centrifuged at 300 g for 5 min. The supernatant was removed after keeping approximately 500–1000 µL samples, and the remaining volume was checked. Samples (10 µL) were placed on glass slides and dried at 40ºC. The cells were fixed using a fixation buffer included in the GenoCTC profiling kit (Genobio Corp.) for 10 min at room temperature. After washing twice with PBS, slides with isolated cells or waste line were mounted using mounting medium containing 1.5 ㎍/mL DAPI (Vector Laboratories Inc., Burlingame, CA, USA; Cat#H-1200) or fluorescence mounting medium (Agilent Technologies, Santa Clara, CA, USA; Cat#S3023), respectively.
