# Supplementary material for: c-MET-positive circulating tumor cells and cell-free DNA as independent prognostic factors in hormone receptor-positive/HER2-negative metastatic breast cancer
Source: Breast Cancer Res. 2024 Jan 18;26:13. doi: 10.1186/s13058-024-01768-y (PMC10797795; doi:10.1186/s13058-024-01768-y)
Supplement: Supplementary file 2 — Additional file 2. Supplementary Fig. S1. The interval between treatment initiation and blood draw grouped by (A) cMET+ CTC, (B) EpCAM+ CTC, and (C) cfDNA concentration. HR, hormone receptor; CTC, circulating tumor cell; cfDNA, cell-free DNA; SD, standard deviation. [file 13058_2024_1768_MOESM2_ESM.docx]

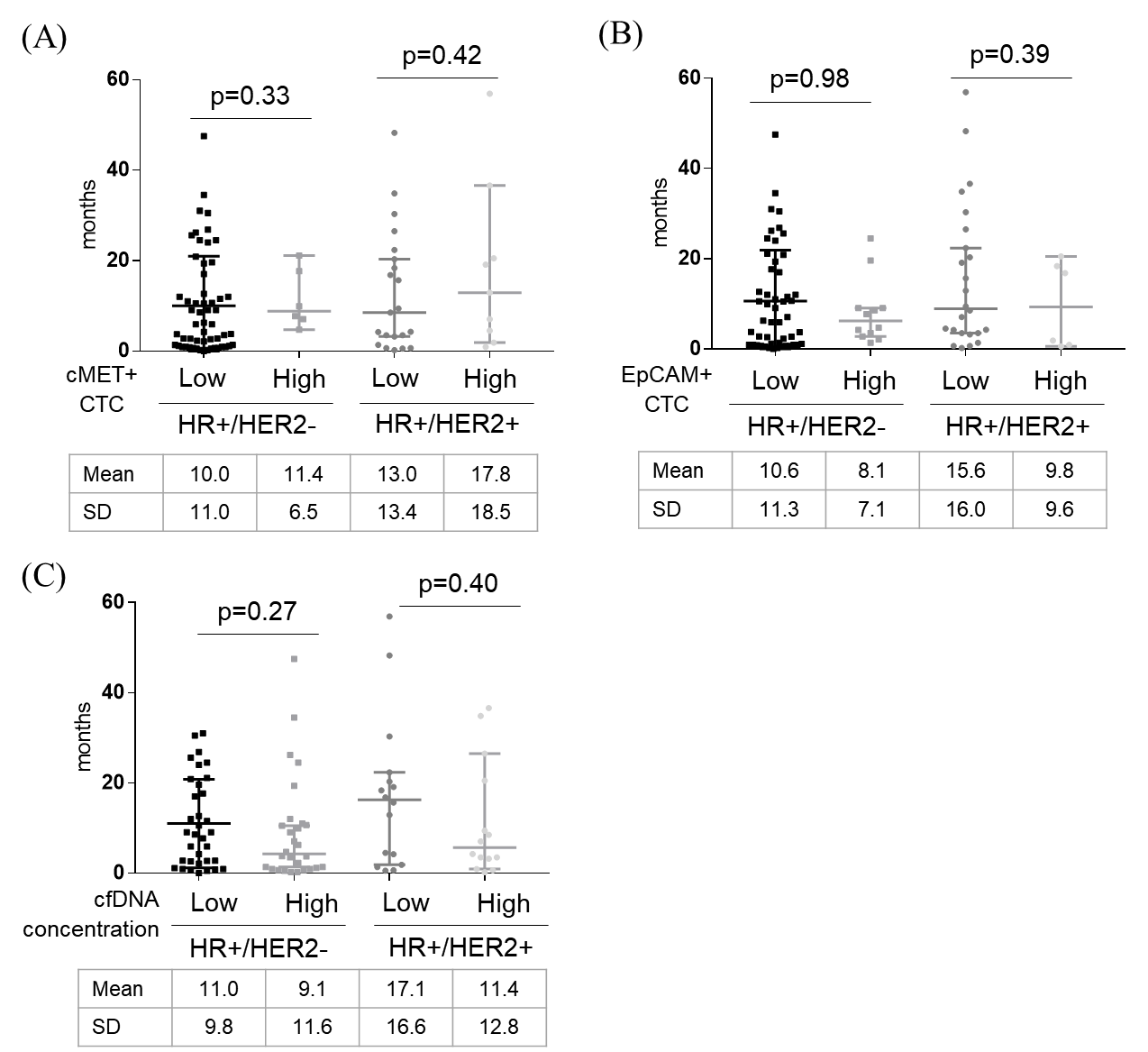


Supplementary Fig. S1 The interval between treatment initiation and blood draw grouped by (A) cMET+ CTC, (B) EpCAM+ CTC, and (C) cfDNA concentration. *HR, hormone receptor;* *CTC, circulating tumor cell; cfDNA, cell-free DNA; SD, standard deviation*
