# Supplementary material for: c-MET-positive circulating tumor cells and cell-free DNA as independent prognostic factors in hormone receptor-positive/HER2-negative metastatic breast cancer
Source: Breast Cancer Res. 2024 Jan 18;26:13. doi: 10.1186/s13058-024-01768-y (PMC10797795; doi:10.1186/s13058-024-01768-y)
Supplement: Supplementary file 3 — Additional file 3. Supplementary Fig. S2. Analytical performance of the c-MET+ CTC assay used in the study. (A) Flow cytometry analysis of SNU5 and MCF7 cells labeled with 2 μg/mL of anti c-MET antibodies. (B) Recovery rates and separation rates of SNU5 and MCF7 cells spiked into buffer (left) and normal human blood (right). Data showed average +/- standard deviation from three experiments. (C) Total number of white blood cells (WBCs) contaminated during CTC enrichment per 4 mL of patient blood. CTC, circulating tumor cells. [file 13058_2024_1768_MOESM3_ESM.docx]

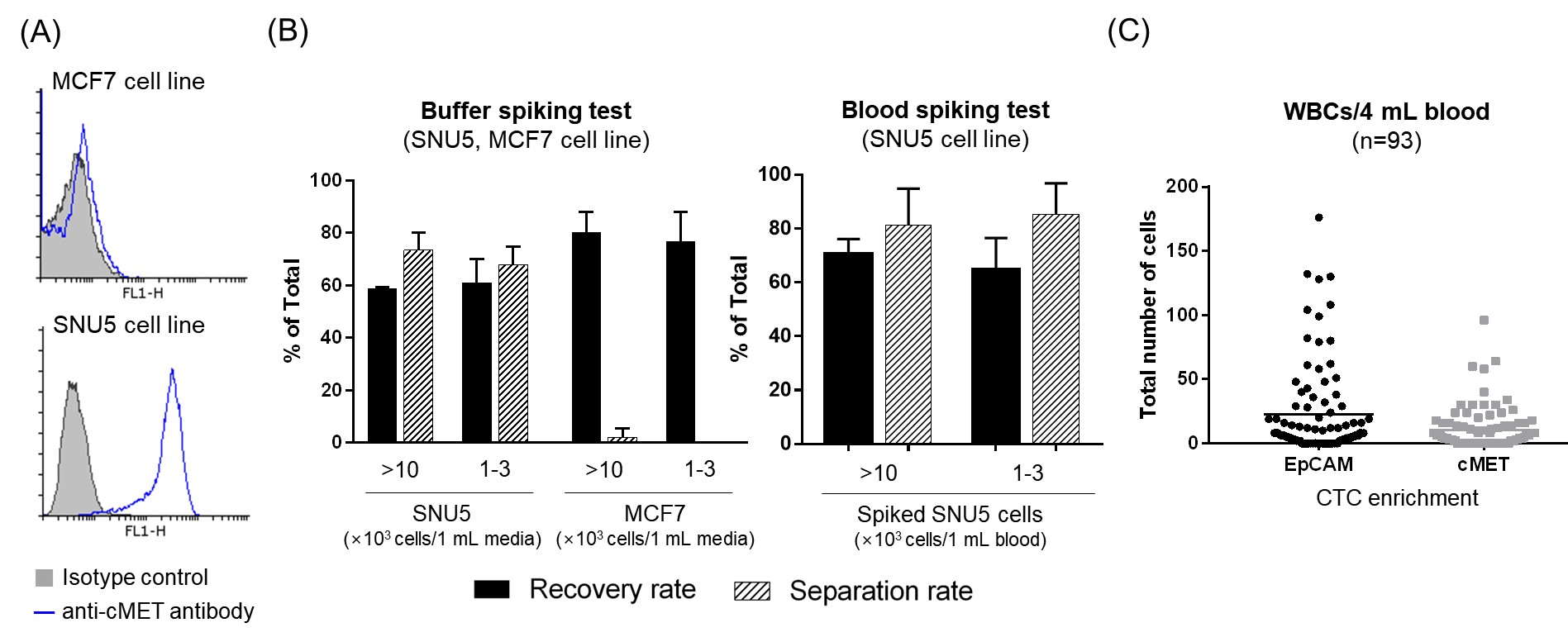


Supplementary Fig. S2 Analytical performance of the c-MET+ CTC assay used in the study. (A) Flow cytometry analysis of SNU5 and MCF7 cells labeled with 2 μg/mL of anti c-MET antibodies. (B) Recovery rates and separation rates of SNU5 and MCF7 cells spiked into buffer (left) and normal human blood (right). Data showed average +/- standard deviation from three experiments. (C) Total number of white blood cells (WBCs) contaminated during CTC enrichment per 4 mL of patient blood. *CTC, circulating tumor cells*
