# Supplementary material for: c-MET-positive circulating tumor cells and cell-free DNA as independent prognostic factors in hormone receptor-positive/HER2-negative metastatic breast cancer
Source: Breast Cancer Res. 2024 Jan 18;26:13. doi: 10.1186/s13058-024-01768-y (PMC10797795; doi:10.1186/s13058-024-01768-y)

Supplementary Figure S3. The number of c-MET+ CTCs in patients with detected CTCs by the site of metastasis. Others refers to cases where the visceral metastatic site was other than the liver.


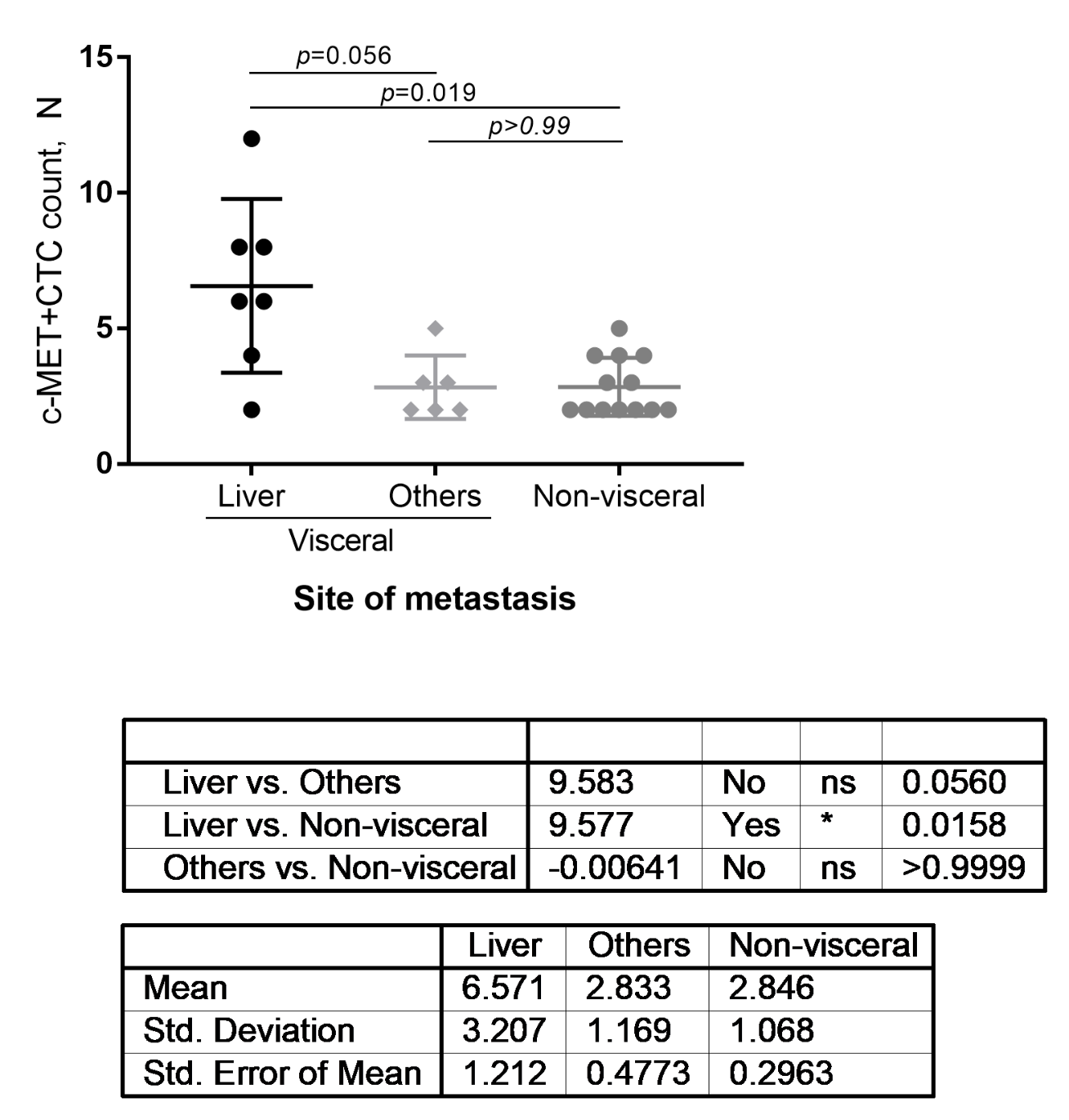

Supplement: Supplementary file 4 — Additional file 4. Supplementary Fig. S3. The number of c-MET+ CTCs in patients with detected CTCs by the site of metastasis. Others refers to cases where the visceral metastatic site was other than the liver. [file 13058_2024_1768_MOESM4_ESM.docx]
