# Supplementary material for: c-MET-positive circulating tumor cells and cell-free DNA as independent prognostic factors in hormone receptor-positive/HER2-negative metastatic breast cancer
Source: Breast Cancer Res. 2024 Jan 18;26:13. doi: 10.1186/s13058-024-01768-y (PMC10797795; doi:10.1186/s13058-024-01768-y)
Supplement: Supplementary file 5 — Additional file 5. Supplementary Table S4. Baseline characteristics of primary and metastatic sites in HR+/HER2- breast cancer. [file 13058_2024_1768_MOESM5_ESM.docx]

Supplementary Table S4. Baseline characteristics of primary and metastatic sites in HR+/HER2- breast cancer

|  |  | Primary breast | | | |  | Metastatic sites | | | |
| --- | --- | --- | --- | --- | --- | --- | --- | --- | --- | --- |
|  |  | Total | Negative  (n=341) | Positive  (n=17) | p-value |  | Total | Negative  (n=21) | Positive  (n=6) | p-value |
| Age at diagnosis | |  |  |  |  |  |  |  |  |  |
|  | 50< | 212 | 202 (59.2) | 10 (58.8) | 1.00 |  | 22 | 16 (76.2) | 6 (100.0) | 0.56 |
|  | 50≥ | 146 | 139 (40.8) | 7 (41.2) |  |  | 5 | 5 (23.8) | 0 (0.0) |  |
| Progesterone receptor | | |  |  |  |  |  |  |  |  |
|  | Negative | 102 | 100 (29.3) | 2 (11.8) | 0.17 |  | 7 | 6 (28.6) | 1 (16.7) | 1.00 |
|  | Positive | 256 | 241 (70.7) | 15 (88.2) |  |  | 20 | 15 (71.4) | 5 (83.3) |  |
| T stage | |  |  |  |  |  |  |  |  |  |
|  | 1 | 159 | 152 (44.6) | 7 (41.2) | 0.78 |  | 9 | 8 (40.0) | 1 (16.7) | 0.26 |
|  | 2 | 179 | 169 (49.6) | 10 (58.8) |  |  | 10 | 8 (40.0) | 2 (33.3) |  |
|  | 3 | 20 | 20 (5.9) | 0 (0.0) |  |  | 6 | 4 (20.0) | 2 (33.3) |  |
|  | 4 | 0 | 0 (0.0) | 0 (0.0) |  |  | 1 | 0 (0.0) | 1 (16.7) |  |
| N stage | |  |  |  |  |  |  |  |  |  |
|  | 0 | 175 | 165 (48.4) | 10 (58.8) | 0.82 |  | 5 | 3 (15.0) | 2 (33.3) | 0.90 |
|  | 1 | 99 | 95 (27.9) | 4 (23.5) |  |  | 12 | 10 (50.0) | 2 (33.3) |  |
|  | 2 | 46 | 45 (13.2) | 1 (5.9) |  |  | 5 | 4 (20.0) | 1 (16.7) |  |
|  | 3 | 38 | 36 (10.6) | 2 (11.8) |  |  | 4 | 3 (15.0) | 1 (16.7) |  |
| Histologic grade | |  |  |  |  |  |  |  |  |  |
|  | 1 | 63 | 58 (17.0) | 5 (29.4) | 0.26 |  | 3 | 3 (16.7) | 0 (0.0) | 0.79 |
|  | 2 | 176 | 171 (50.1) | 5 (29.4) |  |  | 17 | 12 (66.7) | 5 (83.3) |  |
|  | 3 | 112 | 105 (30.8) | 7 (41.2) |  |  | 4 | 3 (16.7) | 1 (16.7) |  |
|  | Unknown | 7 | 7 (2.1) | 0 (0.0) |  |  | 0 | 0 (0.0) | 0 (0.0) |  |
| Nuclear grade | |  |  |  |  |  |  |  |  |  |
|  | 1 | 48 | 45 (13.2) | 3 (17.6) | 0.12 |  | 5 | 4 (22.2) | 1 (16.7) | 1.00 |
|  | 2 | 226 | 219 (64.2) | 7 (41.2) |  |  | 18 | 13 (72.2) | 5 (83.3) |  |
|  | 3 | 73 | 66 (19.4) | 7 (41.2) |  |  | 1 | 1 (5.6) | 0 (0.0) |  |
|  | Unknown | 11 | 11 (3.2) | 0 (0.0) |  |  | 0 | 0 (0.0) | 0 (0.0) |  |
