# Supplementary material for: c-MET-positive circulating tumor cells and cell-free DNA as independent prognostic factors in hormone receptor-positive/HER2-negative metastatic breast cancer
Source: Breast Cancer Res. 2024 Jan 18;26:13. doi: 10.1186/s13058-024-01768-y (PMC10797795; doi:10.1186/s13058-024-01768-y)
Supplement: Supplementary file 6 — Additional file 6. Supplementary Fig. S5. c-MET overexpression in primary breast and metastatic sites in patients with HR+HER2- mBC. (A) Representative immunohistochemical staining intensities for c-MET expression and (B) proportion of c-MET overexpression (positive) in primary breast and metastatic sites. Tumor samples of primary breast and metastatic sites were independently collected and scored.mBC, metastatic breast cancer. [file 13058_2024_1768_MOESM6_ESM.docx]

**
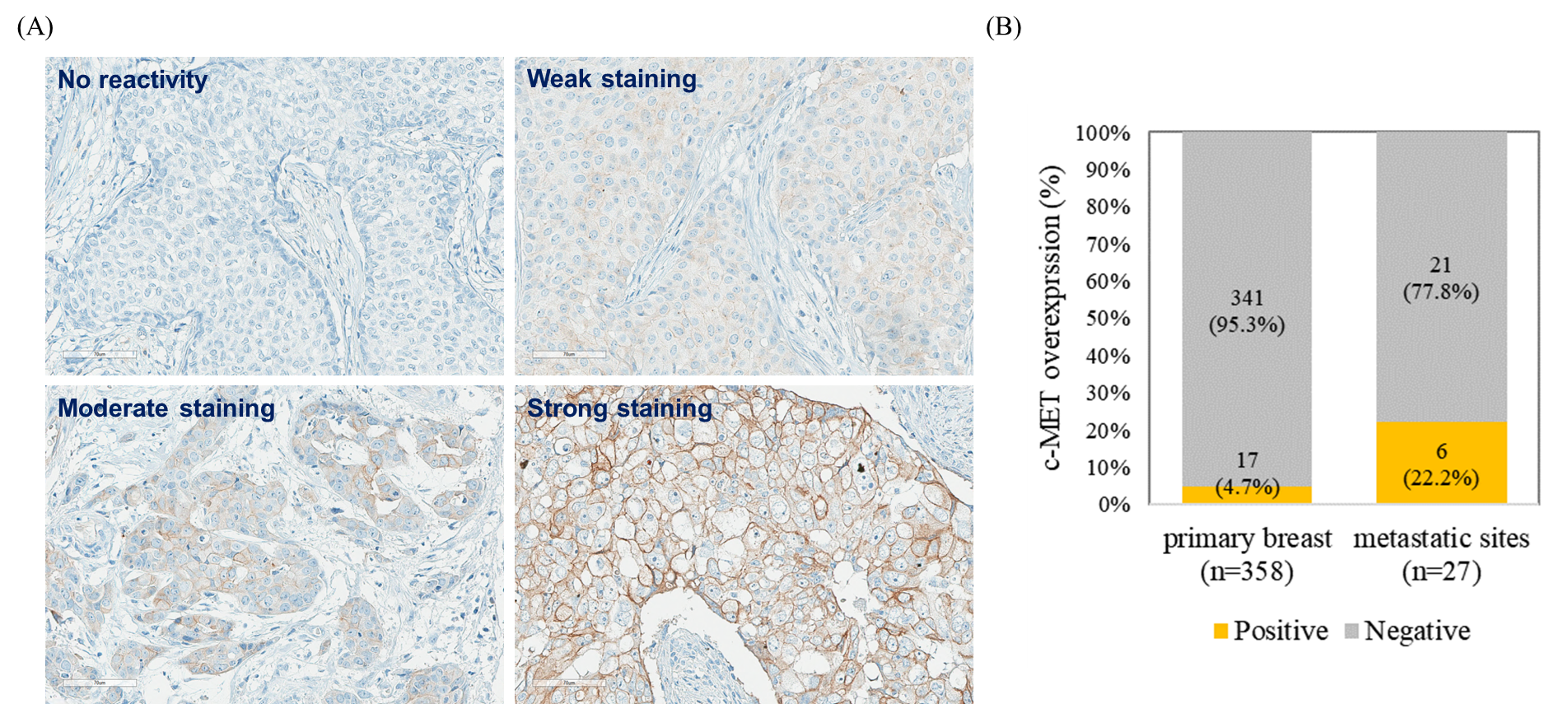
**

Supplementary Fig. S5 c-MET overexpression in primary breast and metastatic sites in patients with HR+HER2- mBC. (A) Representative immunohistochemical staining intensities for c-MET expression and (B) proportion of c-MET overexpression (positive) in primary breast and metastatic sites. Tumor samples of primary breast and metastatic sites were independently collected and scored.*mBC, metastatic breast cancer*
