# Supplementary material for: c-MET-positive circulating tumor cells and cell-free DNA as independent prognostic factors in hormone receptor-positive/HER2-negative metastatic breast cancer
Source: Breast Cancer Res. 2024 Jan 18;26:13. doi: 10.1186/s13058-024-01768-y (PMC10797795; doi:10.1186/s13058-024-01768-y)
Supplement: Supplementary file 7 — Additional file 7. Supplementary Table S6. Summary of previous and present studies on positive rate of c-MET overexpression in breast cancer. [file 13058_2024_1768_MOESM7_ESM.docx]

Supplementary Table S6. Summary of previous and present studies on positive rate of c-MET overexpression in breast cancer

| Ab clone | Disease | Criteria | n | Positive, n | Positive% | References |
| --- | --- | --- | --- | --- | --- | --- |
| SP44 | HR+/HER2- | 0, no staining;  1+, weak or moderate staining in <50% of tumor cells;  2+, incomplete membranous and/or cytoplasmic staining in ≥50% of tumor cells with weak intensity and <50% cells with strong intensity; and  3+, ≥50% of tumor cells with circumferential membranous and/or cytoplasmic staining with strong intensity | 358 | 17 | 4.7 | Present study |
| SP44 | Metastatic  breast cancer, metastatic sites (HR+/HER2-) |  | 27 | 6 | 22.2 | Present study |
| SP44 | HR+/HER2+ |  | 104 | 4 | 3.8 | Present study |
| SP44 | HER2-enriched |  | 98 | 7 | 7.1 | Present study |
| SP44 | TNBC |  | 162 | 22 | 13.6 | Present study |
| SP44 | Breast cancer | >25% of tumor cells with membrane localization were stained | 924 | 386 | 41.8 | [28] |
| SP44 | Metastatic  breast cancer (HR+) | 0, no staining;  1+, weak staining in any amount of tumor cells and moderate staining in <50% of tumor cells;  2+, incomplete membranous and/or cytoplasmic staining in ≥50% tumor cells with moderate intensity and <50% cells with strong intensity; and  3+, ≥ 50% of tumor cells with circumferential membranous and/or cytoplasmic staining with strong intensity | 29 | 12 | 41.4 | [22] |
| SP44 | TNBC |  | 106 | 13 | 12.3 | [39] |
| SP44 | ER+/HER2+ | At least 100 tumor cells per case were evaluated. Staining intensity was scored as 0 (absent), 1 (weak), 2 (intermediate), and 3 (strong) | 78 | 3 | 3.8 | [40] |

*HR, hormone receptor; ER, endocrine receptor; TNBC, triple-negative breast cancer*
