# Supplementary material for: c-MET-positive circulating tumor cells and cell-free DNA as independent prognostic factors in hormone receptor-positive/HER2-negative metastatic breast cancer
Source: Breast Cancer Res. 2024 Jan 18;26:13. doi: 10.1186/s13058-024-01768-y (PMC10797795; doi:10.1186/s13058-024-01768-y)
Supplement: Supplementary file 8 — Additional file 8. Supplementary Fig. S7. Survival analysis using cell-free DNA concentration calculated from the internal control copies of the Droplex PIK3CA Mutation Test Kit, with a cut-off value of 1490 copies/mL plasma in (A) HR+HER2- mBC or (B) HR+HER2+ mBC. mBC, metastatic breast cancer; cfDNA, cell-free DNA. [file 13058_2024_1768_MOESM8_ESM.docx]

Supplementary Figure S7. Survival analysis using cell-free DNA concentration calculated from the internal control copies of the Droplex PIK3CA Mutation Test Kit, with a cut-off value of 1490 copies/mL plasma in (A) HR+HER2- mBC or (B) HR+HER2+ mBC. *mBC, metastatic breast cancer;* *cfDNA, cell-free DNA*

**
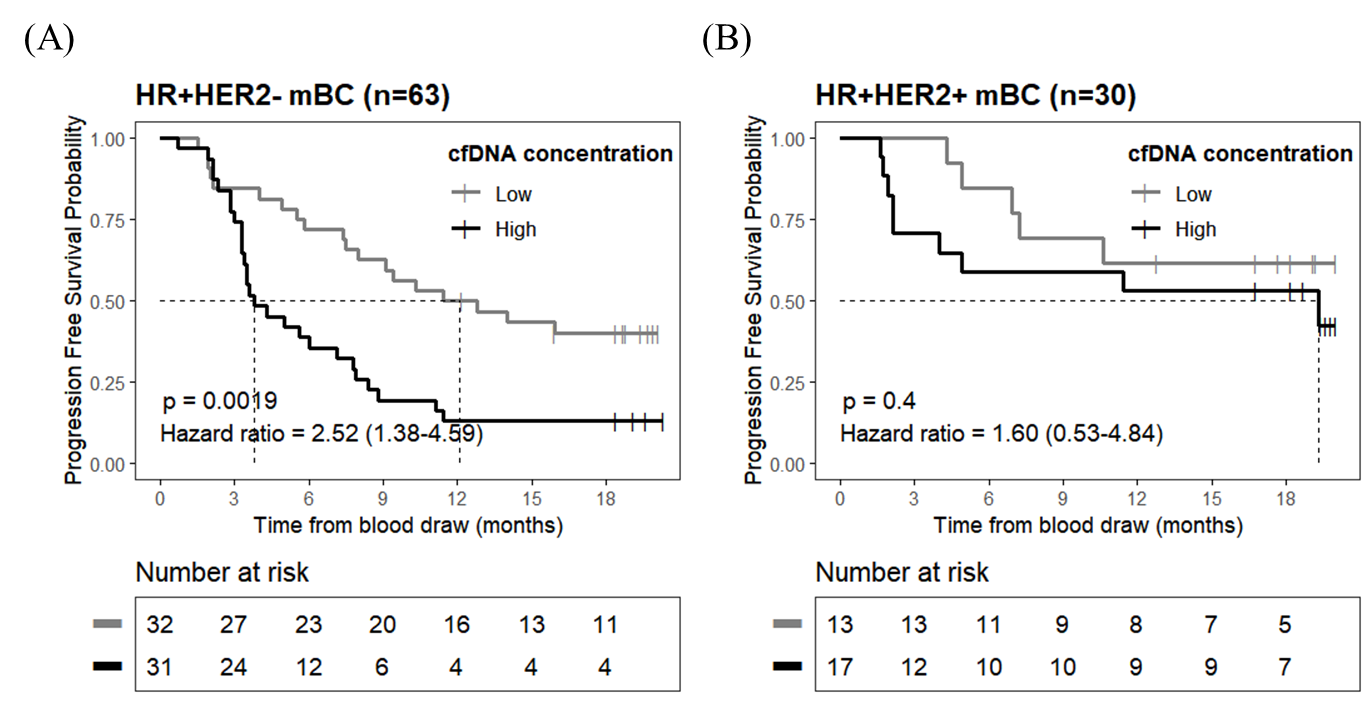
**
