# Supplementary material for: c-MET-positive circulating tumor cells and cell-free DNA as independent prognostic factors in hormone receptor-positive/HER2-negative metastatic breast cancer
Source: Breast Cancer Res. 2024 Jan 18;26:13. doi: 10.1186/s13058-024-01768-y (PMC10797795; doi:10.1186/s13058-024-01768-y)
Supplement: Supplementary file 9 — Additional file 9. Supplementary Table S8. Univariate Cox proportional hazard models for PFS. [file 13058_2024_1768_MOESM9_ESM.docx]

Supplementary Table S8. Univariate Cox proportional hazard models for PFS

|  |  | Univariate | |
| --- | --- | --- | --- |
|  |  | Hazard ratio (95% CI) | p value |
| HR+/HER2- (n=63) | Age (≥50) | 1.1 (0.61-2) | 0.73 |
|  | Visceral metastasis | 1.3 (0.73-2.5) | 0.35 |
|  | Endocrine therapy combined with CDK4/6i | 0.45 (0.24-0.82) | 0.0096 |
|  | Chemotherapy | 2.2 (1.2-4.0) | 0.0074 |
|  | cfDNA concentration (≥1490cp/mL) | 2.5 (1.4-4.5) | 0.0028 |
|  | *ESR1* hotspot mutation | 2.9 (1.3-6.4) | 0.0084 |
|  | *PIK3CA* hotspot mutation | 0.93 (0.39-2.2) | 0.86 |
|  | EpCAM+ CTC (≥4) | 1.4 (0.68-2.8) | 0.38 |
|  | c-MET+ CTC (≥3) | 3.6 (1.5-9.0) | 0.0047 |
| HR+/HER2+ (n=30) | Age (≥50) | 0.85 (0.26-2.7) | 0.78 |
|  | Visceral metastasis | 1.4 (0.42-4.3) | 0.61 |
|  | HER2 targeted therapy | 0.34 (0.1-1.1) | 0.074 |
|  | cfDNA concentration (≥1490cp/mL) | 1.6 (0.51-5.2) | 0.42 |
|  | *ESR1* hotspot mutation | 1.8 (0.63-5.3) | 0.27 |
|  | *PIK3CA* hotspot mutation | 0.96 (0.21-4.4) | 0.96 |
|  | EpCAM+ CTC (≥4) | 2.0 (0.63-6.5) | 0.23 |
|  | c-MET+ CTC (≥3) | 2.4 (0.81-7.2) | 0.12 |

*HR, hormone receptor; cfDNA, cell-free DNA; PFS, progression-free survival; cp/mL, copies per milliliter plasma*
