# Supplementary material for: c-MET-positive circulating tumor cells and cell-free DNA as independent prognostic factors in hormone receptor-positive/HER2-negative metastatic breast cancer
Source: Breast Cancer Res. 2024 Jan 18;26:13. doi: 10.1186/s13058-024-01768-y (PMC10797795; doi:10.1186/s13058-024-01768-y)
Supplement: Supplementary file 10 — Additional file 10. Supplementary Fig. S9. Baseline characteristics, CTC, cfDNA, and PFS data of each patient Tx, Treatment; CTC, circulating tumor cell; Conc., concentration; PFS, progression-free survival. [file 13058_2024_1768_MOESM10_ESM.docx]

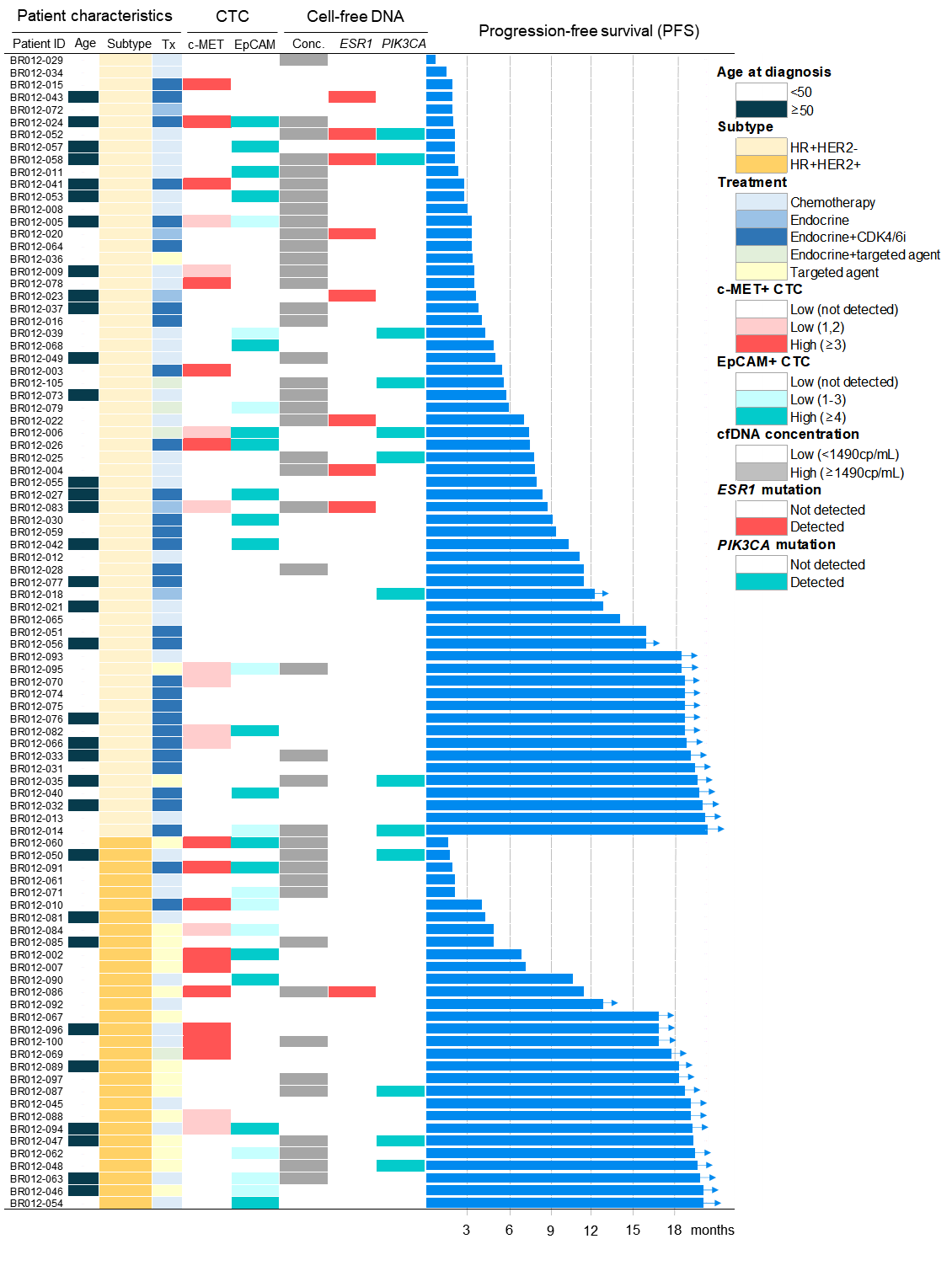


Supplementary Fig. S9 Baseline characteristics, CTC, cfDNA, and PFS data of each patient

*Tx, Treatment; CTC, circulating tumor cell; Conc., concentration; PFS, progression-free survival*
